# Transcriptome sequencing analysis of maize embryonic callus during early redifferentiation

**DOI:** 10.1186/s12864-019-5506-7

**Published:** 2019-02-27

**Authors:** Xiaoling Zhang, Yanli Wang, Yuanyuan Yan, Hua Peng, Yun Long, Yinchao Zhang, Zhou Jiang, Peng Liu, Chaoying Zou, Huanwei Peng, Guangtang Pan, Yaou Shen

**Affiliations:** 1 Key Laboratory of Biology and Genetic Improvement of Maize in Southwest Region, Maize Research Institute, Sichuan Agricultural University, Chengdu, 611130 China; 2 Sichuan Tourism College, Chengdu, 610100 China; 3 Institute of Animal Nutrition, Sichuan Agricultural University, Chengdu, 611130 China

**Keywords:** Maize, Embryonic callus, Redifferentiation, RNA-Seq

## Abstract

**Background:**

Maize is one of the primary crops of genetic manipulation, which provides an excellent means of promoting stress resistance and increasing yield. However, the differences in induction and regeneration capacity of embryonic callus (EC) among various genotypes result in genotypic dependence in genetic transformation.

**Results:**

In this study, embryonic calli of two maize inbred lines with strong redifferentiation capacity and two lines with weak redifferentiation capability were separately subjected to transcriptome sequencing analysis during the early redifferentiation stages (stage I, 1–3 d; stage II, 4–6 d; stage III, 7–9 d) along with their corresponding controls. A total of ~ 654.72 million cDNA clean reads were yielded, and 62.64%~ 69.21% clean reads were mapped to the reference genome for each library. In comparison with the control, the numbers of differentially expressed genes (DEGs) for the four inbred lines identified in the three stages ranged from 1694 to 7193. By analyzing the common and specific DEGs of the four materials, we found that there were 321 upregulated genes and 386 downregulated genes identified in the high-regeneration lines (141 and DH40), whereas 611 upregulated genes and 500 downregulated genes were specifically expressed in the low-regeneration lines (ZYDH381–1 and DH3732). Analysis of the DEG expression patterns indicated a sharp change at stage I in both the high- and low-regeneration lines, which suggested that stage I constitutes a crucial period for EC regeneration. Notably, the specific common DEGs of 141 and DH40 were mainly associated with photosynthesis, porphyrin and chlorophyll metabolism, ribosomes, and plant hormone signal transduction. In contrast, the DEGs in ZYDH381–1 and DH3732 were mainly related to taurine and hypotaurine metabolism, nitrogen metabolism, fatty acid elongation, starch and sucrose metabolism, phenylpropanoid biosynthesis, and plant circadian rhythm. More importantly, *WOX* genes, which have an ancestral role in embryo development in seed plants and promote the regeneration of transformed calli, were specifically upregulated in the two high-regeneration lines.

**Conclusions:**

Our research contributes to the elucidation of molecular regulation during early redifferentiation in the maize embryonic callus.

**Electronic supplementary material:**

The online version of this article (10.1186/s12864-019-5506-7) contains supplementary material, which is available to authorized users.

## Background

Maize (*Zea mays* L.) is a primary global crop supplying the food, feed, and industrial materials industries. Genetic transformation is presently widely used to improve yield and stress resistance and for gene function validation in maize, which largely depend on callus induction and regeneration from maize immature embryos [1–3]. Armstrong et al. [1] classified maize calli into three types, namely, I-, II -, and III-type calli, based on the callus characteristics. Among these types, only the II-type callus, known as embryonic callus, has cell totipotency and the ability to regenerate into whole plants and is therefore widely applied to genetic transformation in maize. Previous studies revealed that the genotype is an important factor that restricts the regeneration of the maize embryonic callus [4–7, 85]. Research on quantitative trait locus (QTL) mapping revealed that the regenerative capability of the embryonic callus is controlled by multiple genes in maize [8, 86].

Several functional genes have been shown to play important roles in callus regeneration in plants. The root stem cell regulators *PLETHORA1 (PLT1)* and *PLETHORA2 (PLT2)* must be activated by *PLETHORA3 (PLT3), PLETHORA5 (PLT5),* and *PLETHORA7 (PLT7)* to establish competent shoot regeneration progenitor cells [11, 12, 14]. A CDK (cyclin-dependent kinase) inhibitor (inhibitor of cyclin-dependent kinase, ICK) has been reported to improve the regenerative capacity of embryonic callus in *Arabidopsis* [20]. Meanwhile, the expression of *WOX5* (*WUSCHEL-related homeobox 5*) in the quiescent center (QC) is considered a marker of *Arabidopsis* root stem cell niche [10]. Whereas, *WUSCHEL (WUS)*, a marker of shoot apical meristem (SAM) identity, together with *CLAVATA1/3 (CLV1/3)*, maintains the SAM stem cell niche through a feedback pathway in *Arabidopsis* [15]. *WUS* also influences shoot stem cell induction activity in the roots [16] and the conversion of root apical meristems (RAMs) to SAMs depending on the exogenous plant growth hormones applied in vitro [17]. In addition, as an AP2/ERF transcription factor, *WIND1* (*WOUND INDUCED DEDIFFERENTIATION 1*) was proven to upregulate the expression of ESR1 (*ENHANCER OF SHOOT REGEENRATION 1*) gene that encodes another AP2/ERF transcription factor, promoting *Arabidopsis* shoot regeneration [9, 13]. *SOMATIC EMBRYOGENESIS RECEPTOR KINASE 1 (SERK1),* which is involved in the acquisition of embryogenic competence in plant tissue culture, is strongly expressed during the early stages of somatic embryogenesis in *Arabidopsis* [18, 19]. The downregulation of multiple CDK inhibitor *ICK/KRP* genes additively enhances both the shoot and root regeneration abilities of root-derived callus in *Arabidopsis*, indicating that CDK activity is a major factor for in vitro organogenesis [20]. Nishimura et al. reported that a ferredoxin-nitrite reductase (NiR) was responsible for rice regeneration ability [83]. In a recent study, *WOX2* (*WUSCHEL-related homeobox 2*) and *BBM* (*Baby Boom)* genes were together introduced into maize by genetic transformation, resulting in the increased number of resistant seedlings regenerated from the transformed immature embryos [79]. In our latest study, 40 candidate genes were identified as being associated with the regenerative capacity of embryonic callus in maize, with regulators in cell fate determination, auxin transport, seed germination, or embryo development [85]. The present study was aimed at revealing the regulatory mechanisms associated with the early redifferentiation of embryonic callus by using the transcriptome data of four maize inbred lines with different regeneration capacities.

## Results

### Phenotypic evaluation of the four inbred lines

**Fig. 1:**
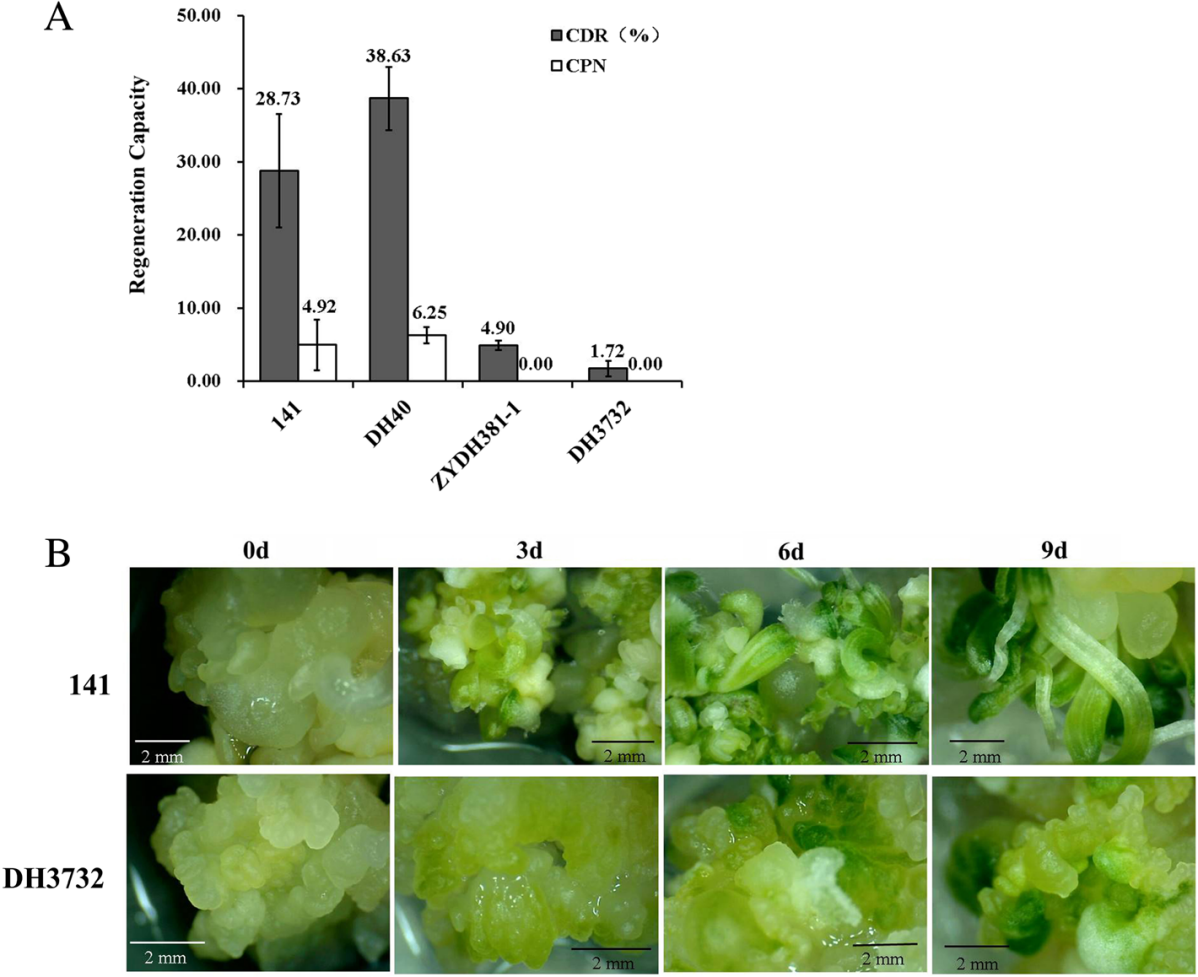
Phenotypic evaluation of the four inbred lines. **a** Regeneration ability of the EC of the four inbred lines; **b** The growth status of the EC of maize inbred lines 141 and DH3732 at 0 d, 3 d, 6 d, and 9 d

The EC regeneration capacities of the four lines were investigated in our previous study [85]. The CDR (callus differentiating rate) and CPN (callus plantlet number) of inbred lines 141 and DH40 were much higher than DH3732 and ZYDH381–1 (Fig. 1a) [85]. For the high-regeneration materials (141 and DH40), some small adventitious buds grew from the callus at 3 d, a mass of adventitious buds were generated at 6 d, and little plantlets formed at 9 d. For the low-regeneration materials (DH3732 and ZYDH381–1), only some calli became green after 6 d, and no adventitious bud formation was observed during the whole process (Fig. 1b). Based on the morphological features of 141 and DH40, the early redifferentiation of EC was divided into three stages: stage I (1–3 d), stage II (4–6 d), and stage III (7–9 d).

### Transcriptome sequencing of maize EC

In total, the transcriptome sequencing of 48 libraries produced ~ 654.72 million clean reads after filtering low-quality reads, adaptor-polluted reads, and high unknown base (N) reads. The number of clean reads ranged from 13,342,096 to 14,067,230 (average = 13,640,022) among the samples. The results of the base composition and quality analysis showed that the clean reads had a good base composition (the T curve was in accordance with the A curve, and the G curve was in accordance with the C curve), and the percentage of low-quality reads was lower than 20% (Additional file 1: Figure S1). The B73 genome was used as the reference for read mapping, and HISAT was used to map the reads against the reference. Finally, only 60.73 to 67.38% of the remaining reads could be uniquely mapped onto the reference genome sequence (Table 1).

**Table 1:** Statistics of all the samples mapped to the reference genome

Line	Stage	Biological replicates	All reads	Mapped Reads	Unmapped Reads	Unique Mapped Rades	Mapping Rates	Unique Mapping Rates
141	Control	1	13,822,187	8,714,174	5,108,013	8,485,703	0.6304	0.6139
		2	13,434,657	8,598,351	4,836,306	8,360,218	0.6400	0.6223
		3	13,985,300	8,979,156	5,006,144	8,740,197	0.6420	0.6250
	Stage I	1	14,055,149	9,202,394	4,852,755	8,943,796	0.6547	0.6363
		2	14,044,846	9,286,325	4,758,521	9,028,947	0.6612	0.6429
		3	13,449,932	8,819,316	4,630,616	8,575,390	0.6557	0.6376
	Stage II	1	13,366,341	8,603,256	4,763,085	8,378,170	0.6437	0.6268
		2	14,067,230	9,026,700	5,040,530	8,778,827	0.6417	0.6241
		3	13,404,850	8,783,565	4,621,285	8,552,222	0.6553	0.6380
	Stage III	1	14,011,138	9,139,636	4,871,502	8,885,358	0.6523	0.6342
		2	13,992,015	9,025,007	4,967,008	8,771,520	0.6450	0.6269
		3	13,404,219	8,755,804	4,648,415	8,510,385	0.6532	0.6349
ZYDH381–1	Control	1	13,535,081	9,264,675	4,270,406	8,992,364	0.6845	0.6644
		2	13,446,968	9,102,739	4,344,229	8,836,735	0.6769	0.6572
		3	13,477,135	9,024,884	4,452,251	8,770,579	0.6696	0.6508
	Stage I	1	14,017,567	9,617,028	4,400,539	9,372,156	0.6861	0.6686
		2	13,638,752	9,364,693	4,274,059	9,126,774	0.6866	0.6692
		3	13,507,384	9,348,406	4,158,978	9,101,693	0.6921	0.6738
	Stage II	1	13,412,867	9,156,535	4,256,332	8,916,334	0.6827	0.6648
		2	13,447,590	9,225,042	4,222,548	8,993,871	0.6860	0.6688
		3	13,452,005	9,246,867	4,205,138	9,004,531	0.6874	0.6694
	Stage III	1	13,348,510	9,087,187	4,261,323	8,850,995	0.6808	0.6631
		2	13,454,704	9,138,751	4,315,953	8,897,604	0.6792	0.6613
		3	13,364,828	9,002,084	4,362,744	8,775,990	0.6736	0.6566
DH3732	Control	1	13,542,727	8,787,059	4,755,668	8,532,583	0.6488	0.6300
		2	13,440,273	8,418,935	5,021,338	8,162,067	0.6264	0.6073
		3	13,467,285	8,502,174	4,965,111	8,235,774	0.6313	0.6115
	Stage I	1	13,950,754	8,748,280	5,202,474	8,507,545	0.6271	0.6098
		2	13,913,447	8,765,080	5,148,367	8,512,786	0.6300	0.6118
		3	13,900,865	8,794,870	5,105,995	8,543,188	0.6327	0.6146
	Stage II	1	13,380,869	8,553,535	4,827,334	8,319,669	0.6392	0.6218
		2	13,915,518	8,791,565	5,123,953	8,541,320	0.6318	0.6138
		3	13,416,949	8,499,351	4,917,598	8,265,621	0.6335	0.6161
	Stage III	1	13,389,978	8,582,279	4,807,699	8,333,240	0.6409	0.6223
		2	13,876,459	8,882,088	4,994,371	8,616,149	0.6401	0.6209
		3	13,867,073	8,887,087	4,979,986	8,630,474	0.6409	0.6224
DH40	Control	1	13,449,919	8,689,301	4,760,618	8,487,275	0.6460	0.6310
		2	13,540,509	8,643,739	4,896,770	8,422,063	0.6384	0.6220
		3	13,482,696	8,542,803	4,939,893	8,337,800	0.6336	0.6184
	Stage I	1	13,749,465	8,820,722	4,928,743	8,596,240	0.6415	0.6252
		2	13,703,965	8,704,100	4,999,865	8,483,808	0.6352	0.6191
		3	13,776,438	8,854,412	4,922,026	8,623,368	0.6427	0.6260
	Stage II	1	13,978,916	9,059,933	4,918,983	8,822,962	0.6481	0.6312
		2	13,342,096	8,623,085	4,719,011	8,403,633	0.6463	0.6299
		3	13,401,358	8,706,297	4,695,061	8,477,048	0.6497	0.6326
	Stage III	1	13,629,650	8,803,902	4,825,748	8,570,618	0.6459	0.6288
		2	13,824,878	8,802,559	5,022,319	8,575,593	0.6367	0.6203
		3	13,637,727	8,892,013	4,745,714	8,651,254	0.6520	0.6344

Gene expression levels were calculated using FPKM and were estimated using Cufflinks [24–26]. A correlation value between biological replicates for each of the stages was calculated according to the FPKM result. The Pearson’s correlations were mostly higher than 0.90 (Additional file 2: Figure S2), indicating good repeatability of the sequencing data.

### Reliability validation of DEG expression via qRT-PCR

**Fig. 2:**
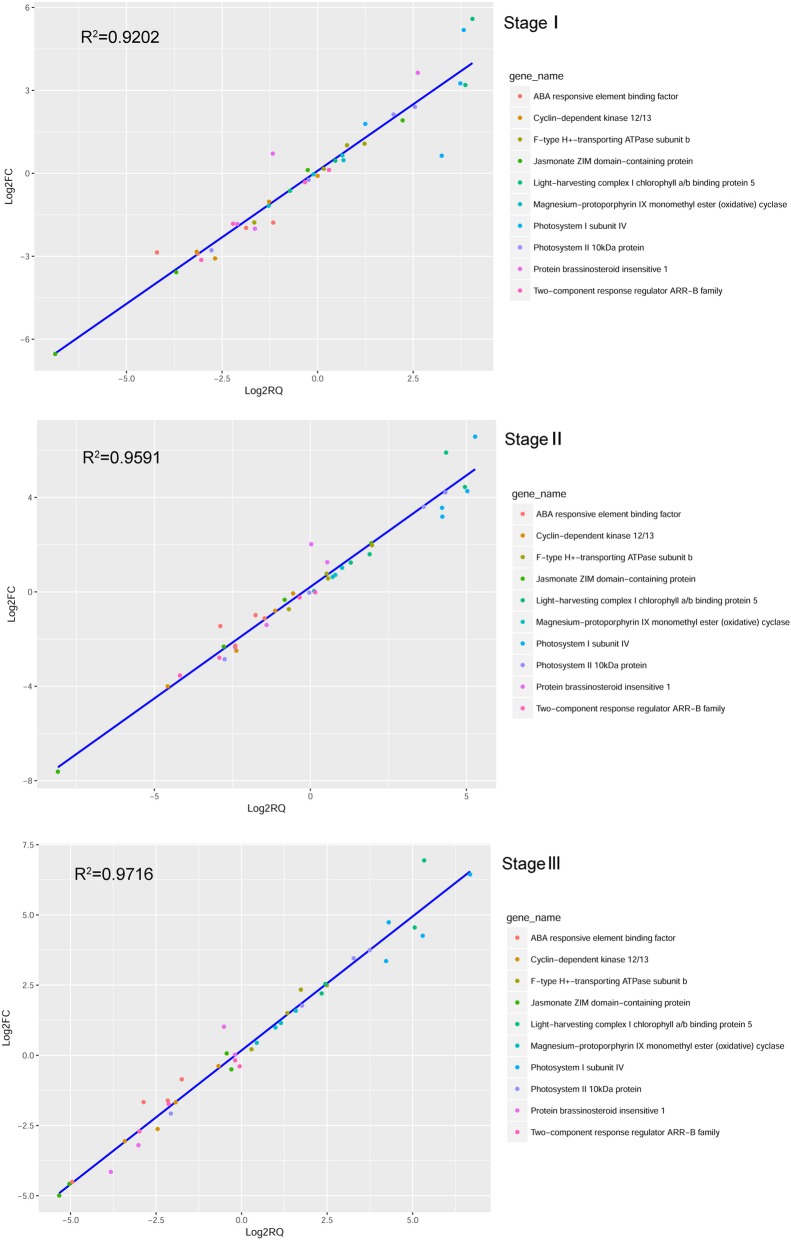
Correlation of the differential expression ratio between qRT-PCR and RNA-Seq in the three stages

Ten DEGs involved in different biological processes (photosynthesis, plant hormone signal transduction, and protein phosphorylation) were randomly selected for qRT-PCR to validate the reliability of the transcriptome sequencing data. The results showed that the Pearson’s correlation coefficients between the data generated from the two platforms were all higher than 0.9 (*R*^*2*^ > 0.90) for the three stages (Fig. 2), indicating that the RNA-Seq data were reliable.

### Specific and common DEGs in the lines with different regeneration capacities

**Fig. 3:**
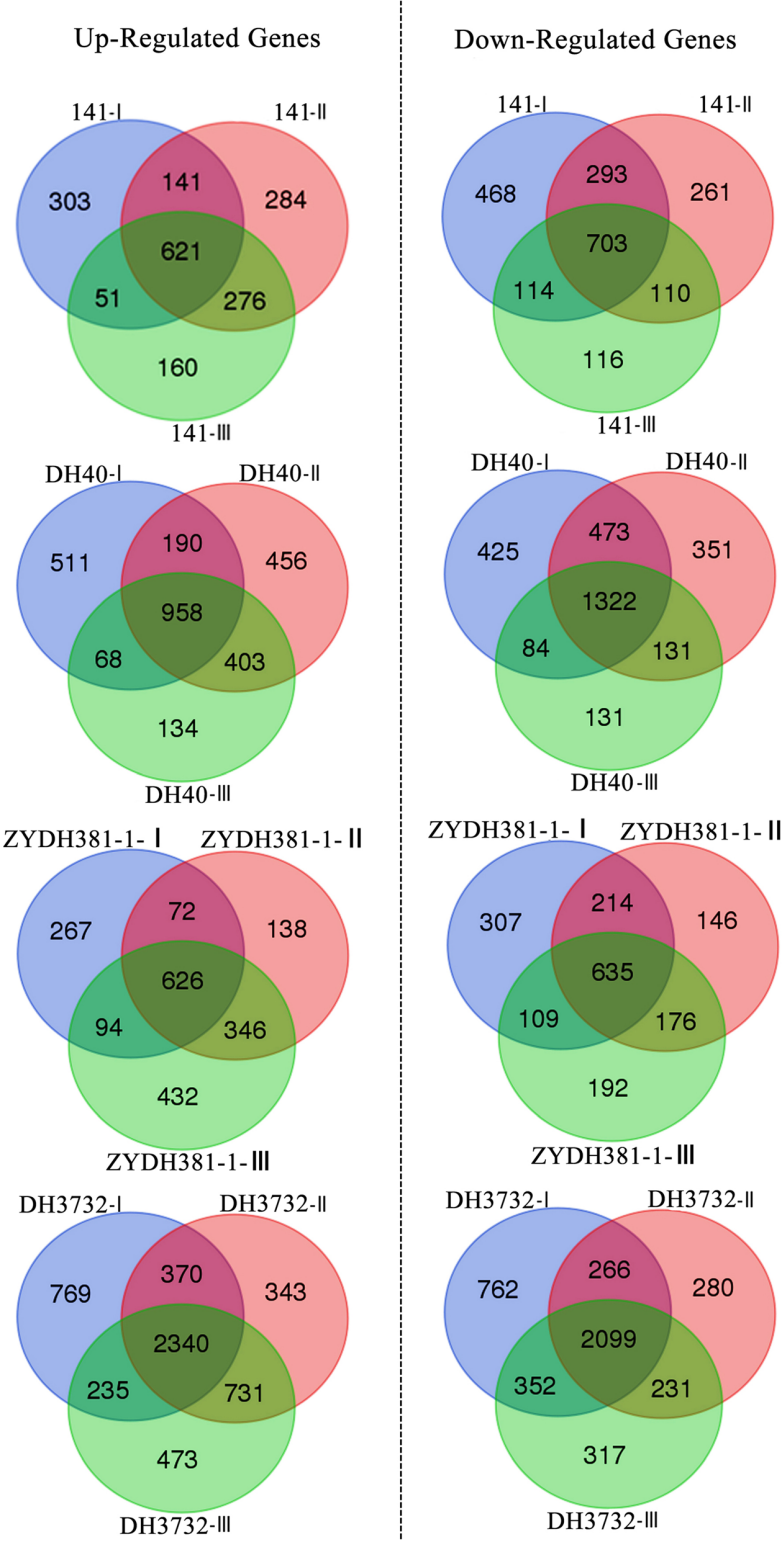
Venn diagram of the DEGs for each inbred line

**Fig. 4:**
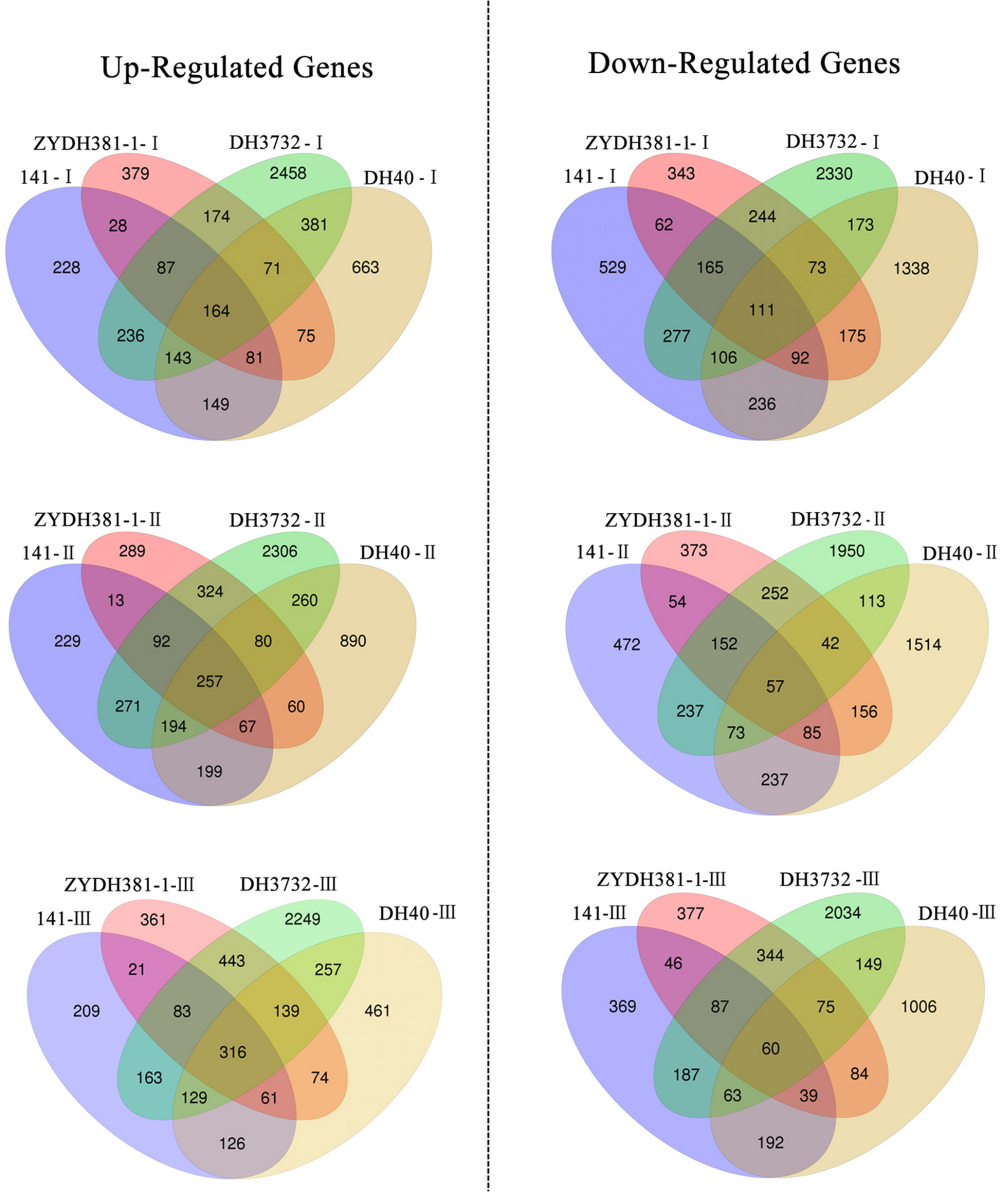
Venn diagram of the DEGs between each inbred line

To gain insight into the regulatory network of embryonic callus redifferentiation, we analyzed the similarities and differences in the DEGs among the four maize inbred lines. For each line, the common DEGs among all the stages were much higher than the specific DEGs detected by each of the stages (Fig. 3), indicating that only a small number of the DEGs were involved in the early redifferentiation capability of EC. We further focused on the common DEGs shared by the high-regeneration lines (141 and DH40), which showed differential expression patterns in each of the low-regeneration lines (ZYDH381–1 and DH3732) during the process of redifferentiation (Fig. 4). These DEGs were accordingly called specific common DEGs in the high-regeneration lines, which were positively correlated with EC regeneration. Conversely, the specific common DEGs in the low-regeneration lines probably exerted inhibitory effects on EC regeneration**.** Detailed information on these specific common DEGs is shown in Additional file 3: Tables S3 and S4. In total, 385 (149 up and 236 down), 436 (199 up and 237 down), and 318 (126 up and 192 down) specific common DEGs were detected at stage I, stage II, and stage III, respectively, amounting to 707 DEGs in the three stages. In contrast, in the low-regeneration lines, a total of 1111 specific common DEGs were identified (Fig. 4). Specifically, 418 (174 up and 244 down), 576 (324 up and 252 down), and 787 (443 up and 344 down) DEGs were expressed at stage I, stage II, and stage III, respectively. According to the adjusted *P*-value, *Zm00001d019518* (Photosystem I reaction center subunit IV A), *Zm00001d042178* (Photosystem II reaction center psb28 protein), and *Zm00001d039687* (Photosystem I reaction center subunit XI chloroplastic) were the most significant DEGs in 141 and DH40 (Table 2). Conversely, *Zm00001d007049* (cysteine proteinases superfamily protein), *Zm00001d044122* (dihydroflavonol-4-reductase), and *Zm00001d024281* (polyamine oxidase 1) were the most significant DEGs in ZYDH381–1 and DH3732 (Table 3).

**Table 2:** The most differentially expressed specific common DEGs of the two high-regeneration lines

GeneID	Gene description	Expression_ ratios											
		141			DH40			DH3732			ZYDH381–1		
		I/CK	II/CK	III/CK	I/CK	II/CK	III/CK	I/CK	II/CK	III/CK	I/CK	II/CK	III/CK
*Zm00001d019518*	Photosystem I reaction center subunit IV A	3.25	4.27	4.74	5.18	6.58	6.44	0.64	3.56	4.26	1.79	3.18	3.36
*Zm00001d042178*	Photosystem II reaction center psb28 protein	3.15	3.73	3.98	4.95	5.76	5.70	−2.39	0.00	0.33	0.72	1.80	2.15
*Zm00001d039687*	Photosystem I reaction center subunit XI chloroplastic	3.01	3.88	4.12	3.99	5.21	4.90	0.20	0.30	−0.02	0.00	0.34	1.09
*Zm00001d009929*	Beta-propeller domain of methanol dehydrogenase type%3B Beta-propeller domains of methanol dehydrogenase type	3.24	4.73	5.20	3.90	5.85	5.84	1.27	4.15	5.23	1.77	3.82	4.42
*Zm00001d028756*	Uncharacterized protein	3.32	3.54	3.90	3.82	4.15	4.29	0.59	2.21	2.75	0.77	1.58	1.76
*Zm00001d049048*	Heat shock protein binding protein	3.53	3.78	3.56	3.12	3.57	3.56	0.19	1.05	1.13	−0.03	1.08	1.30
*Zm00001d014564*	Oxygen-evolving enhancer protein 1–1 chloroplastic	2.82	3.50	3.71	3.48	4.53	4.31	−0.76	1.07	1.78	−0.13	0.73	1.01
*Zm00001d034283*	Photosystem I reaction center subunit III	2.69	3.61	3.83	3.13	4.79	4.59	−4.13	−1.72	−1.16	−0.81	0.15	0.26
*Zm00001d025848*	Plastid transcriptionally active 5	2.63	4.03	4.04	3.06	4.53	4.57	1.24	2.68	3.63	1.19	2.80	2.95
*Zm00001d016991*	Uncharacterized protein	2.42	3.01	3.29	3.29	4.16	3.91	−1.12	0.89	1.32	0.89	1.72	2.02
*novel_G001848*	Uncharacterized protein	−5.86	−5.83	−5.77	−5.90	−0.68	− 1.79	−8.19	−7.18	− 1.96	−0.28	0.04	−0.22
*Zm00001d007623*	Patatin-like protein 2	−5.22	−2.49	−2.90	−3.00	−0.49	− 1.67	2.73	3.24	2.85	−0.13	−0.21	0.78
*novel_G001959*	Hypothetical protein	−4.63	− 1.57	− 1.08	− 2.11	− 4.63	− 1.76	− 1.49	− 1.62	− 0.83	− 0.15	− 0.67	−0.69
*Zm00001d015427*	V-type proton atpase catalytic subunit A	−4.32	− 4.30	− 4.22	−3.57	−3.80	− 3.37	− 1.12	− 1.04	−1.08	− 0.46	− 0.46	− 0.49
*novel_G001626*	Translation initiation factor IF-2	− 3.79	− 3.76	− 0.21	− 2.87	− 0.36	− 3.48	− 2.44	− 2.27	− 0.12	− 0.18	− 0.08	−2.72
*novel_G002132*	Serine/threonine-protein phosphatase 7 long form homolog	−2.83	− 3.52	−0.46	− 4.61	−6.74	− 4.70	− 1.69	− 1.42	− 1.59	0.34	0.15	0.00
*Zm00001d031785*	Receptor-like protein kinase 4	−2.55	− 3.21	− 3.18	− 3.00	− 2.85	− 2.25	− 0.48	− 0.67	− 1.26	− 1.14	−2.00	− 1.21
*Zm00001d007604*	Patatin-like protein 2	−2.31	−3.17	−2.24	− 3.28	− 2.13	−2.40	4.32	3.43	4.32	− 0.86	− 1.34	− 0.78
*Zm00001d046634*	Uncharacterized protein	− 1.66	−2.24	− 3.07	− 2.85	− 3.03	− 1.70	−0.99	− 0.50	− 2.15	− 0.89	−0.81	− 1.27
*Zm00001d050133*	Homoserine dehydrogenase	−0.91	−5.52	−0.95	− 2.36	− 6.41	− 1.67	− 0.86	− 1.75	−0.79	− 0.27	−0.84	− 0.90

**Table 3:** The most differentially expressed specific common DEGs of the two low-regeneration lines

GeneID	Gene description	Expression_ ratios											
		ZYDH381–1			DH3732			141			DH40		
		I/CK	II/CK	III/CK	I/CK	II/CK	III/CK	I/CK	II/CK	III/CK	I/CK	II/CK	III/CK
*Zm00001d007049*	Cysteine proteinases superfamily protein	5.33	5.02	4.56	8.00	6.03	4.70	1.80	0.96	0.91	−0.06	−0.45	− 1.04
*Zm00001d044122*	Dihydroflavonol-4-reductase	4.17	6.05	6.16	5.10	4.54	4.79	1.22	2.64	3.07	2.15	3.75	3.40
*Zm00001d024281*	Polyamine oxidase1	3.88	3.47	3.98	4.68	3.90	3.88	0.58	0.24	0.87	3.06	3.25	2.27
*Zm00001d011461*	Putative uncharacterized protein	3.70	4.09	5.07	3.74	5.86	6.02	0.23	1.72	1.13	−0.34	0.66	1.32
*Zm00001d045254*	Anthocyanidin 5,3-O-glucosyltransferase	3.39	4.22	4.43	3.45	3.70	3.28	2.12	2.01	0.34	3.40	2.60	1.50
*Zm00001d043242*	Early nodulin 20	3.27	2.65	3.88	2.25	4.02	4.73	0.34	−0.43	−0.16	1.88	1.65	0.40
*Zm00001d038718*	Hemoglobin2	2.98	3.03	3.54	3.88	4.70	4.82	0.79	1.21	1.03	0.21	0.21	0.24
*Zm00001d012231*	Amino acid permease 6	2.49	3.43	4.61	2.61	4.71	4.66	0.17	−0.44	0.54	1.36	0.98	0.75
*novel_G000032*	Opie1 putative gag protein	−4.74	−4.74	−4.81	−10.44	−10.25	−10.40	0.00	0.00	0.00	0.00	0.00	0.00
*Zm00001d019704*	Uncharacterized protein	−3.87	−3.89	−3.07	−2.34	−2.89	−3.05	−2.61	−2.66	−2.09	−3.60	−3.26	− 1.10
*Zm00001d048693*	ATA15 protein	−3.63	− 3.19	− 3.37	−3.23	− 1.86	− 2.05	− 2.24	− 1.30	−0.95	−2.74	−1.09	− 1.29
*Zm00001d039762*	ARM repeat superfamily protein	−3.49	− 3.51	− 3.56	−3.66	− 3.47	−1.06	− 0.43	−2.40	−0.36	− 0.90	−1.75	− 1.37
*Zm00001d038049*	Lichenase-2	−3.02	−4.12	− 3.88	−6.32	−5.18	−5.38	−1.25	− 1.01	− 0.80	0.49	1.70	1.03
*Zm00001d023387*	Hypothetical protein	−2.91	−3.16	−3.01	−2.83	− 3.29	−3.59	−1.98	− 3.74	−0.37	− 2.49	−3.75	−1.96
*Zm00001d022022*	Zinc finger protein 7	− 2.80	−3.06	−3.11	− 5.51	−4.72	− 4.86	0.16	0.16	0.34	0.61	0.28	0.00

### Expression patterns of specific common DEGs during redifferentiation

**Fig. 5:**
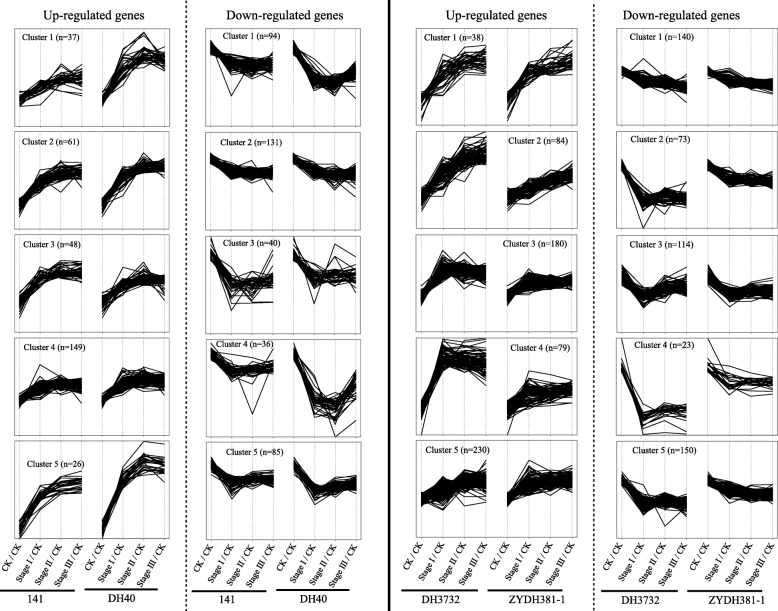
The expression patterns of specific common genes in high (low) regeneration lines

To assess the expression patterns of these specific common DEGs during early redifferentiation, we conducted a K-means approach using ExpressCluster software, as described in Ge et al. [84]. The expression patterns of the up- or downregulated specific common DEGs were classified into five clusters (Fig. 5). Interestingly, the majority of the clusters in both the high- and low-regeneration lines indicated sharp changes in DEGs at stage I, but more moderate changes at stage II and III, which suggested that stage I played a key role in the regeneration of EC. Somatic embryogenesis, which controls the future morphogenesis of plantlets, mainly occurred at stage I, and thus we supposed that these DEGs were particularly important for embryoid formation. Specifically, the upregulated DEGs in cluster 5 for the high-regeneration lines exhibited a greater change than the other clusters (Additional file 4: Table S5), and the majority of these DEGs were related to photosynthesis, such as *Zm00001d019518* (photosystem I subunit IV) and *Zm00001d042178* (photosystem II 13 kDa protein). Furthermore, the downregulated DEGs in cluster 3 for the high-regeneration lines showed identical and significant declines in stage I (Additional file 4: Table S6), and these DEGs were involved in the cellular protein modification process (*Zm00001d042551*, integrin-linked kinase), primary metabolic process (*Zm00001d008952*, endoglucanase; *Zm00001d014244*, alpha-L-fucosidase), and defense response (*Zm00001d034461*, indole-3-glycerol-phosphate lyase). For the DEGs in the low-regeneration lines, the upregulated DEGs in cluster 1 continuously increased during the three stages (Additional file 4: Table S7), and most of these genes were related to the metabolic process, such as *Zm00001d044122* (dihydroflavonol-4-reductase) and *Zm00001d018161* (ferredoxin-nitrite reductase). These downregulated specific common DEGs of cluster 3 displayed consistent expression trends in both the lines, with a sharp reduction in stage I and a slight increase in stage II and III (Additional file 4: Table S8), which included *Zm00001d017913* (somatic embryogenesis receptor kinase 1), *Zm00001d048647* (transcription factor MYB108-like), and *Zm00001d032376* (disease resistance protein RPM1). These DEGs may account for the low regeneration capacity in ZYDH381–1 and DH3732.

### GO analysis for specific common DEGs

To obtain functional annotations of the genes involved in EC regeneration capacity, we carried out GO analysis for the specific common DEGs. Both the upregulated and downregulated genes of the specific common DEGs of 141 and DH40 were categorized into three functions: Biological Process (BP), Cellular Component (CC), and Molecular Function (MF). As shown in Additional file 5: Figure S3, many terms of plant growth and development were significantly enriched in these biological processes, and the significantly enriched entries (FDR ≤ 0.05 or *P*-value ≤0.05) are displayed in Additional file 6: Tables S9–S11. Among the upregulated genes, 265 were assigned with functional annotations involved in 332 terms for BP, 65 terms for CC, and 13 terms for MF. BP included photosynthesis, glyceraldehyde-3-phosphate metabolic process, isopentenyl diphosphate biosynthetic process, cellular aldehyde metabolic process, phospholipid biosynthetic process, biosynthetic process and metabolic process of pigment, shoot and system form morphogenesis, the process of embryonic development, and seed development. CC involved chloroplast and chloroplast part, plasmids and plasmid part, and NAD(P)H dehydrogenase complex. Meanwhile, MF included the structural constituent of ribosome, transcription factor activity and core RNA polymerase binding, and chlorophyll binding. In the downregulated genes, 247 were annotated with functions, including seven terms in BP (regulation of jasmonic acid-mediated signaling pathway, response to wounding, regulation of defense response, regulation of response to stress, regulation of signaling and signal transduction, and regulation of cell communication), two in CC (integral component of membrane, intrinsic component of membrane), and four in MF (transcription co-repressor activity, transcription cofactor activity, transcription factor activity and transcription factor binding, and protein binding).

The specific common DEGs of ZYDH381–1 and DH3732 with 611 upregulated genes and 500 downregulated genes were also divided into three functional categories of BP, CC, and MF (Additional file 7: Figure S4), and the significant GO terms of enrichment (FDR ≤ 0.05) are shown in Additional file 8: Tables S12–S14. For the upregulated genes, a total of 458 DEGs were assigned functional annotations, including 13 terms in BP (nucleosome assembly and nucleosome organization, protein-DNA complex assembly and protein-DNA complex subunit organization, DNA conformation change and DNA packaging, oxidation–reduction process, monocarboxylic acid biosynthetic process, and fatty acid biosynthetic process), 16 in CC (protein-DNA complex, nucleosome, chromosomal part, chromosome, and cell wall), and 14 in MF (protein heterodimerization activity, protein dimerization activity, beta-glucosidase activity, galactosidase activity, and beta-galactosidase activity). As for the downregulated genes, 314 DEGs had functional annotations with 47 GO terms in BP (regulation of nucleobase-containing compound metabolic process, regulation of RNA metabolic process, regulation of nitrogen compound metabolic process, regulation of macromolecule biosynthetic process, auxin-activated signaling pathway, oligosaccharide biosynthetic process, the metabolic process and biosynthetic process of strigolactone, and cellular response to light stimulus), one in CC (nucleus), and six in MF (DNA binding, nucleic acid binding transcription factor activity, polysaccharide binding, and photoreceptor activity).

### Pathways enriched by specific common DEGs

To further understand the functional role of the DEGs in the process of EC regeneration, KEGG pathway analysis of the specific common DEGs of 141 and DH40 was conducted (Additional file 9: Table S15). In stage I, 66 upregulated DEGs and 85 downregulated DEGs with functional annotations were involved in 40 and 58 metabolic pathways, respectively. In stage II, 91 upregulated DEGs and 87 downregulated DEGs with functional annotations were included in 61 and 51 metabolic pathways, respectively. In stage III, 56 upregulated DEGs and 69 downregulated DEGs with functional annotations affected 44 and 51 metabolic pathways, respectively. Photosynthesis, ribosome, and porphyrin and chlorophyll metabolism were three of the most significantly enriched upregulated pathways and were shared by the three stages of EC regeneration, indicating the conserved and important roles of these three metabolic pathways in the process of EC regeneration. Moreover, plant hormone signal transduction was the most significantly enriched pathway for the downregulated DEGs shared by the three stages of EC regeneration, suggesting that the decreased expression of the genes in this pathway positively influences EC regeneration. The significantly enriched pathways for the specific common DEGs of 141 and DH40 in stage I, II, and III samples are listed in Table 4.

**Table 4:** List of significant enrichment pathways for specific common DEGs of 141 and DH40 (*P*-value ≤0.05)

Pathway term	Pathway ID	DEGs tested	*P* value	Q value
Stage I sample				
Up-regulated DEGs				
Photosynthesis	ko00195	12	1.89E-13	7.54E-12
Ribosome	ko03010	18	3.53E-09	7.06E-08
Porphyrin and chlorophyll metabolism	ko00860	6	9.85E-05	1.31E-03
Photosynthesis - antenna proteins	ko00196	3	5.89E-04	5.89E-03
Oxidative phosphorylation	ko00190	5	8.53E-03	6.83E-02
Glycine, serine and threonine metabolism	ko00260	4	1.06E-02	7.06E-02
Limonene and pinene degradation	ko00903	2	4.56E-02	2.61E-01
Down-regulated DEGs				
Plant hormone signal transduction	ko04075	17	1.06E-04	6.54E-03
Cutin, suberine and wax biosynthesis	ko00073	5	9.67E-04	3.00E-02
Linoleic acid metabolism	ko00591	2	2.01E-02	3.78E-01
Benzoxazinoid biosynthesis	ko00402	2	3.05E-02	3.78E-01
Stage II sample				
Up-regulated DEGs				
Porphyrin and chlorophyll metabolism	ko00860	8	9.28E-06	5.94E-04
Ribosome	ko03010	14	2.46E-04	7.89E-03
Photosynthesis	ko00195	5	9.83E-04	2.10E-02
Diterpenoid biosynthesis	ko00904	3	1.10E-02	1.41E-01
C5-Branched dibasic acid metabolism	ko00660	2	1.41E-02	1.50E-01
Ubiquinone and other terpenoid-quinone biosynthesis	ko00130	3	1.66E-02	1.52E-01
Stilbenoid, diarylheptanoid and gingerol biosynthesis	ko00945	4	2.04E-02	1.63E-01
Glycine, serine and threonine metabolism	ko00260	4	3.17E-02	2.26E-01
Down-regulated DEGs				
Plant hormone signal transduction	ko04075	25	1.35E-09	7.56E-08
Plant-pathogen interaction	ko04626	12	2.42E-03	6.77E-02
Pentose and glucuronate interconversions	ko00040	4	4.84E-02	6.77E-01
Stage III sample				
Up-regulated DEGs				
Porphyrin and chlorophyll metabolism	ko00860	5	4.52E-04	1.40E-02
Ribosome	ko03010	10	5.94E-04	1.40E-02
Photosynthesis	ko00195	4	1.25E-03	1.96E-02
Stilbenoid, diarylheptanoid and gingerol biosynthesis	ko00945	4	3.90E-03	4.59E-02
Ubiquinone and other terpenoid-quinone biosynthesis	ko00130	2	4.38E-02	3.84E-01
Peroxisome	ko04146	3	4.90E-02	3.84E-01
Down-regulated DEGs				
Plant hormone signal transduction	ko04075	19	1.31E-07	6.80E-06
Other glycan degradation	ko00511	4	1.32E-02	3.43E-01
Flavonoid biosynthesis	ko00941	3	3.64E-02	3.59E-01
Glycerolipid metabolism	ko00561	3	3.77E-02	3.59E-01
Glycerophospholipid metabolism	ko00564	4	4.89E-02	3.59E-01

Pathways enriched in the specific common DEGs of ZYDH381–1 and DH3732 are indicated in Additional file 10: Table S16. In stage I, 69 upregulated DEGs and 79 downregulated DEGs were involved in 71 and 62 metabolic pathways, respectively. In stage II, 118 upregulated DEGs and 100 downregulated DEGs were included in 78 and 76 metabolic pathways, respectively. In stage III samples, 178 upregulated DEGs and 112 downregulated affected 90 and 71 metabolic pathways, respectively. For upregulated DEGs, taurine and hypotaurine metabolism was the only significantly enriched pathway that was shared by the three stages of EC regeneration, and nitrogen metabolism was the only enriched pathway shared by stages I and II. In addition, 10 significant enrichment pathways (fatty acid elongation, starch and sucrose metabolism, phenylpropanoid biosynthesis, glycosaminoglycan degradation, sphingolipid metabolism, galactose metabolism, glycosphingolipid biosynthesis-ganglio series, phenylalanine metabolism, other glycan degradation, and stilbenoid, diarylheptanoid, and gingerol biosynthesis) were shared by stages II and III. Among these, numerous DEGs were involved in the pathway of phenylpropanoid biosynthesis, which is the most significant metabolic pathway enriched in stage III. For the downregulated DEGs, seven pathways (plant circadian rhythm, carbon metabolism, valine, leucine and isoleucine degradation, plant hormone signal transduction, glycolysis/gluconeogenesis, propanoate metabolism, and cyanoamino acid metabolism) were identified in the three stages of EC regeneration, among which the plant circadian rhythm was the most significant pathway in stages I and II and the second most significant pathway in stage III, whereas plant hormone signal transduction was the most significant pathway in stage III. These significant pathways common to the three stages might have a repressive effect on EC regeneration (Table 5).

**Table 5:** List of significant enrichment pathways for specific common DEGs of ZYDH381–1 and DH3732 (*P*-value ≤0.05)

Pathway term	Pathway ID	DEGs tested	P value	Q value
Stage I sample				
Up-regulated DEGs				
Taurine and hypotaurine metabolism	ko00430	3	7.34E-05	5.29E-03
Nitrogen metabolism	ko00910	3	7.36E-03	2.65E-01
Flavonoid biosynthesis	ko00941	3	3.51E-02	6.46E-01
Down-regulated DEGs				
Circadian rhythm – plant	ko04712	8	4.70E-04	2.99E-02
Carbon metabolism	ko01200	11	9.05E-04	2.99E-02
Valine, leucine and isoleucine degradation	ko00280	4	2.98E-03	6.56E-02
Plant hormone signal transduction	ko04075	11	2.11E-02	3.22E-01
Caffeine metabolism	ko00232	1	2.44E-02	3.22E-01
Glycolysis / Gluconeogenesis	ko00010	5	3.87E-02	3.74E-01
Propanoate metabolism	ko00640	2	4.50E-02	3.74E-01
Cyanoamino acid metabolism	ko00460	3	4.86E-02	3.74E-01
Stage II sample				
Up-regulated DEGs				
Fatty acid elongation	ko00062	5	8.82E-04	2.05E-02
Starch and sucrose metabolism	ko00500	14	9.09E-04	2.05E-02
Phenylpropanoid biosynthesis	ko00940	11	1.02E-03	2.05E-02
Glycosaminoglycan degradation	ko00531	5	1.04E-03	2.05E-02
Sphingolipid metabolism	ko00600	6	3.06E-03	3.94E-02
Galactose metabolism	ko00052	7	3.20E-03	3.94E-02
Glycosphingolipid biosynthesis - ganglio series	ko00604	4	4.14E-03	3.94E-02
Phenylalanine metabolism	ko00360	4	4.35E-03	3.94E-02
Other glycan degradation	ko00511	6	4.49E-03	3.94E-02
Taurine and hypotaurine metabolism	ko00430	2	8.80E-03	6.95E-02
Nitrogen metabolism	ko00910	3	3.07E-02	2.13E-01
beta-Alanine metabolism	ko00410	3	3.39E-02	2.13E-01
Photosynthesis - antenna proteins	ko00196	2	3.51E-02	2.13E-01
Stilbenoid, diarylheptanoid and gingerol biosynthesis	ko00945	4	4.49E-02	2.53E-01
RNA polymerase	ko03020	3	4.96E-02	2.61E-01
Down-regulated DEGs				
Circadian rhythm – plant	ko04712	10	9.18E-05	3.84E-03
Valine, leucine and isoleucine degradation	ko00280	6	9.85E-05	3.84E-03
Plant hormone signal transduction	ko04075	17	4.45E-04	1.16E-02
Propanoate metabolism	ko00640	3	8.61E-03	1.13E-01
Glycolysis / Gluconeogenesis	ko00010	7	8.68E-03	1.13E-01
Alanine, aspartate and glutamate metabolism	ko00250	4	1.04E-02	1.16E-01
Fatty acid degradation	ko00071	3	1.78E-02	1.48E-01
Cyanoamino acid metabolism	ko00460	4	1.91E-02	1.48E-01
Nitrogen metabolism	ko00910	3	2.06E-02	1.48E-01
Glycerolipid metabolism	ko00561	4	2.21E-02	1.48E-01
Arginine biosynthesis	ko00220	3	2.27E-02	1.48E-01
Tyrosine metabolism	ko00350	3	2.67E-02	1.60E-01
Carbon metabolism	ko01200	9	3.64E-02	2.03E-01
Stage III sample				
Up-regulated DEGs				
Phenylpropanoid biosynthesis	ko00940	22	3.77E-08	3.54E-06
Sphingolipid metabolism	ko00600	9	3.48E-04	1.45E-02
Starch and sucrose metabolism	ko00500	19	5.78E-04	1.45E-02
Other glycan degradation	ko00511	9	6.18E-04	1.45E-02
Glycosaminoglycan degradation	ko00531	6	1.17E-03	2.21E-02
Glycosphingolipid biosynthesis - ganglio series	ko00604	5	3.28E-03	4.67E-02
Phenylalanine metabolism	ko00360	5	3.48E-03	4.67E-02
Cutin, suberine and wax biosynthesis	ko00073	6	4.27E-03	4.71E-02
Porphyrin and chlorophyll metabolism	ko00860	7	4.51E-03	4.71E-02
Galactose metabolism	ko00052	8	9.75E-03	8.64E-02
Cyanoamino acid metabolism	ko00460	6	1.01E-02	8.64E-02
Stilbenoid, diarylheptanoid and gingerol biosynthesis	ko00945	6	1.69E-02	1.33E-01
Taurine and hypotaurine metabolism	ko00430	2	1.99E-02	1.43E-01
Terpenoid backbone biosynthesis	ko00900	6	2.13E-02	1.43E-01
Fatty acid elongation	ko00062	4	2.74E-02	1.72E-01
Brassinosteroid biosynthesis	ko00905	3	2.96E-02	1.74E-01
Flavonoid biosynthesis	ko00941	5	4.07E-02	2.25E-01
Down-regulated DEGs				
Plant hormone signal transduction	ko04075	20	8.00E-05	5.92E-03
Circadian rhythm – plant	ko04712	10	2.52E-04	9.31E-03
Glycolysis / Gluconeogenesis	ko00010	9	1.26E-03	2.82E-02
Valine, leucine and isoleucine degradation	ko00280	5	1.52E-03	2.82E-02
Propanoate metabolism	ko00640	3	1.20E-02	1.36E-01
Carbon metabolism	ko01200	11	1.29E-02	1.36E-01
Fatty acid degradation	ko00071	3	2.45E-02	1.98E-01
Nitrogen metabolism	ko00910	3	2.81E-02	1.98E-01
Cyanoamino acid metabolism	ko00460	4	2.83E-02	1.98E-01
Caffeine metabolism	ko00232	1	3.42E-02	1.98E-01
Pyruvate metabolism	ko00620	5	3.45E-02	1.98E-01
Anthocyanin biosynthesis	ko00942	2	3.48E-02	1.98E-01

### Specific common DEGs involved in EC regeneration

The early redifferentiation of EC is a process of somatic embryogenesis and adventitious shoot regeneration. By comparing the DEGs of the four inbred lines, we found that a number of genes were involved in processes of photosynthesis, hormone signaling transduction, cell cycle, embryo and meristem initiation, circadian rhythm of plant, and phenylpropanoid biosynthesis. Based on the functional annotations, we identified some significantgenes closely related to callus regeneration (listed in Additional file 11: Table S17).

**Fig. 6:**
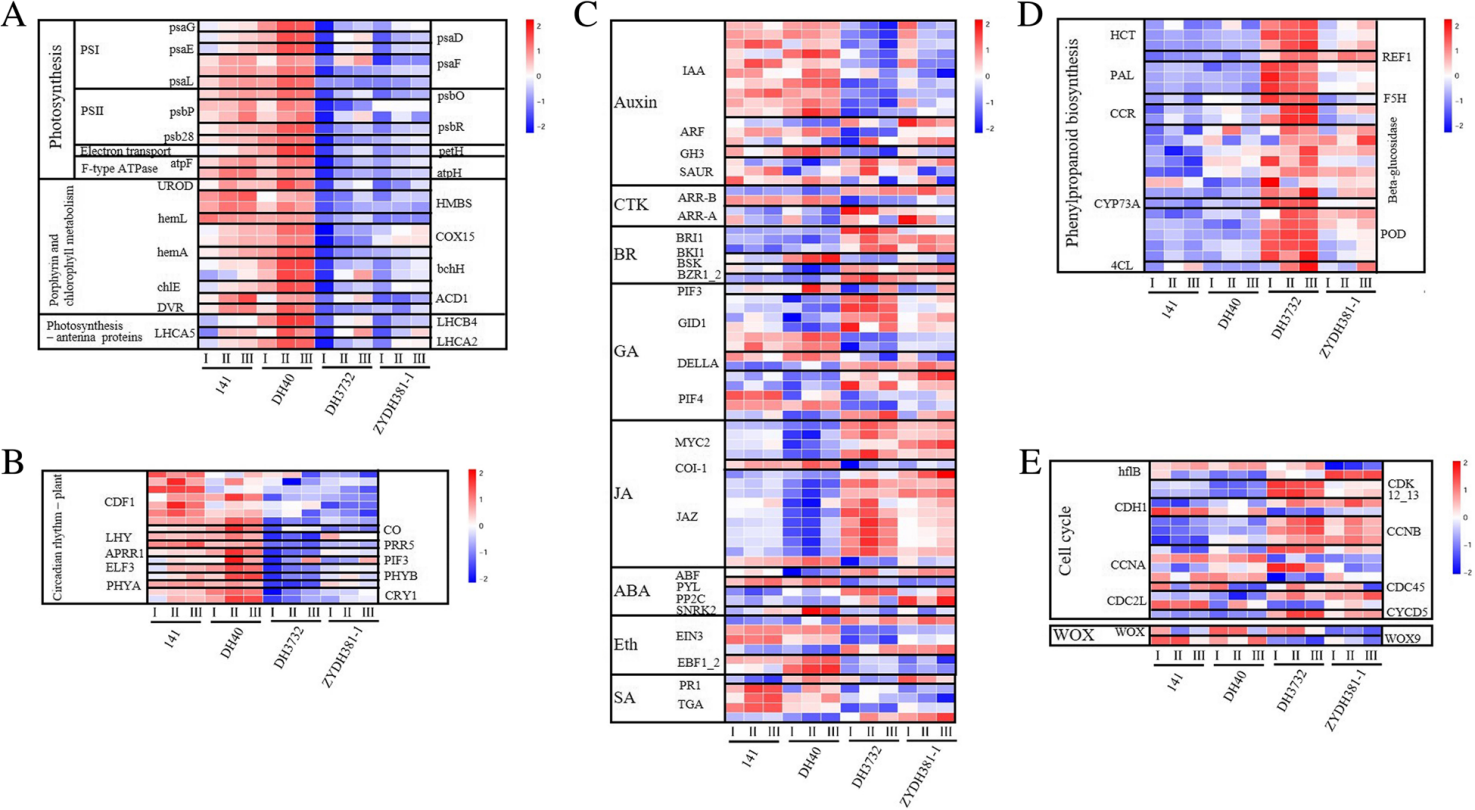
Relative expression patterns of the DEGs involved in tissue regeneration. **a** The relative expression pattern of DEGs involved in photosynthesis mechanism; **b** The relative expression pattern of DEGs involved in plant circadian rhythm; **c** The relative expression pattern of DEGs involved in plant hormone signal transduction mechanisms; **d** The relative expression pattern of DEGs involved in phenylpropanoid biosynthesis; **e** The relative expression pattern of DEGs related to cell cycling and WUSCHEL homeobox protein

Photosynthesis mechanism: Photosynthesis is essential for the survival and development of plantlets. According to the functional annotations of the specific common DEGs of 141 and DH40, 14 upregulated genes were involved in the pathway of photosynthesis (Fig. 6a and Additional file 12: Figure S5). In addition, 12 genes were involved in porphyrin and chlorophyll metabolism (Fig. 6a and Additional file 13: Figure S6). Moreover, three DEGs including *Zm00001d021906*, *Zm00001d018157*, and *Zm00001d006587*, were involved in the pathway of photosynthetic antenna proteins, all of which were only upregulated in stage I (Fig. 6a and Additional file 14: Figure S7). We speculated that photosynthesis could effectively promote the regeneration of embryogenic callus in the two high-regeneration lines.

Plant circadian rhythm: A total of 17 specifically downregulated DEGs in both ZYDH381–1 and DH3732 were associated with plant circadian rhythm (Fig. 6b). Of these DEGs, seven were annotated as Dof zinc finger protein DOF5.5 (CDF1), and two DEGs (*Zm00001d003477* and *Zm00001d016915*) were annotated as cryptochrome 1 (CRY1). Especially *CRY1* and *ELF3,* which respond to blue light and therefore affect the photomorphogenesis of the plant, the other DEGs respond to red light and regulate plant cell elongation and flowering (Additional file 15: Figure S8).

Plant hormone signal transduction mechanisms: Plant hormones play an important role in EC regeneration [53–60, 80]. Based on the specific common DEGs of 141 and DH40, a total of 29 downregulated genes were involved in hormone signal transduction (Fig. 6c). Of these, nine genes were annotated as jasmonate ZIM domain-containing protein (JAZ), and four genes were annotated as gibberellin receptor GID1 (GID1). Some specifically upregulated genes in 141 and DH40 were also related to the hormone transduction signal (Fig. 6c), including ABA receptor PYR/PYL family (PYL) (*Zm00001d016105*) and GID1 (*Zm00001d007908, Zm00001d022320*). Notably, JAZ and MYC2, as important factors of jasmonic acid signal transduction, were found to play critical roles in senescence and the plant stress response [88] (Additional file 16: Figure S9). Furthermore, GID1 and PIF4, which participate in the signal transduction pathway of gibberellin, have important functions in stem growth and the induction of seed germination (Additional file 16: Figure S9). For the specific common DEGs of ZYDH381–1 and DH3732, a total of 30 downregulated genes were related to plant hormone signal transduction (Fig. 6c). Among them, 11 genes were annotated as auxin-responsive protein IAA (IAA). *SAUR* (*Zm00001d017397*, *Zm00001d018200*), auxin responsive *GH3* gene family (*GH3*) (*Zm00001d010697*), and *ARF* (*Zm00001d014690*) were identified in the downregulated genes of the specific common DEGs of ZYDH381–1 and DH3732. These genes are all involved in auxin signal transduction, which is important for cell enlargement and plant growth (Additional file 17: Figure S10). Additionally, EIN3 and EBF play roles in ethylene signal transduction, which is related to senescence and fruit ripening (Additional file 17: Figure S10). Ten specifically upregulated genes in ZYDH381–1 and DH3732 were also involved in ethylene signal transduction. In summary, these DEGs participate in the signal transduction pathways of several hormones, including auxin, cytokinin (CTK), gibberellin (GA), jasmonic acid (JA), and brassinosteroid (BR), indicating that the cross-talking of these hormones plays important roles in EC regeneration.

Phenylpropanoid biosynthesis: A total of 24 upregulated DEGs were related to the metabolic pathway of phenylpropanoid biosynthesis, specifically in ZYDH381–1 and DH3732 (Fig. 6d and Additional file 18: Figure S11). Of these, beta-glucosidase is a glycoside hydrolase enzyme that participates in various cell functions, such as the catalysis of disaccharide and oligosaccharide bonds, catalysis of cell wall components, and the activation of phytohormones [27–31]. POD protects tissues and cells from oxidative damage by catalyzing the reduction of H_2_O_2_, and its accumulation accelerates the browning of the callus [28]. PAL catalyzes the nonoxidative elimination of ammonia from L-Phe to yield *trans*-cinnamate in the first step of the phenylpropanoid pathway, which plays an essential role in plant development and the stress response [29–31]. In the two lines with poor EC regeneration, the DEGs involved in phenylpropanoid biosynthesis were all increased at the same stage, which suggested that phenylpropanoid biosynthesis negatively influenced EC redifferentiation by accelerating callus browning.

The DEGs related to cell cycling: For the specific common DEGs of 141 and DH40, one upregulated gene and two downregulated genes were related to cell cycle regulation (Fig. 6e), including *cell division protease FtsH* (*Zm00001d036371*) and *cyclin-dependent kinase 12/13* (*Zm00001d022041*, *Zm00001d042285*). In addition, 10 upregulated genes and four downregulated genes were assigned to this category for specific common DEGs of ZYDH381–1 and DH3732 (Fig. 6e). Of these, cyclin-dependent kinase 12/13 (CDK12_13) belongs to CDKs, which are the core regulators of the cell cycle and are assumed to control cell differentiation and proliferation in response to phytohormone signals [32, 33]. Furthermore, cyclin A (CCNA) and cyclin B (CCNB) are both members of the cyclin family, which regulates CDK activity [34]. Cell division protease FtsH (FtsH) is a part of ATP-dependent proteases, which function in protein quality control and regulation [35].

Moreover, *Zm00001d035535,* which encodes a putative WUSCHEL homeobox protein (WOX), was specifically upregulated in 141 and DH40 at all the three stages, whereas *Zm00001d042821,* annotated as WUSCHEL-related homeobox 9-like (WOX9), was significantly downregulated in ZYDH381–1 and DH3732 in stage III (Fig. 6e). WOX family proteins have been proved to regulate embryo development and callus regeneration [78, 82]. Therefore, the cross-talk of these DEGs related to the cell cycle and WOX could positively influence embryonic callus regeneration.

Cluster analysis of the relative expressions of these DEGs (Additional file 19: Figure S12) showed that inbred lines 141 and DH40 were clustered into one group, while ZYDH381–1 and DH3732 as well as the three stages of each lines were separately clustered together. Furthermore, stage II and III were categorized into one class, whereas stage I was alone. These results indicated that the expression levels of these DEGs between the high- and low-regeneration lines were significantly distinct. The responses of these DEGs were rapid, being largest in the first stage and smaller in the following stages.

## Discussion

### Effect of light on callus regeneration

Light, which is closely related to the gain of energy and metabolic processes, is usually an important factor affecting growth, organogenesis, and the formation of plant products, including both primary and secondary metabolites [36]. A number of reports have mentioned that plant regeneration from a callus requires light and that plant regeneration derived from a callus is effectively increased in light compared to the dark [36–41]. Rikiishi et al. reported that light probably controls shoot regeneration from calli by modifying cytokinin levels and/or responses, and blue light signals act in the photoinhibition of shoot regeneration in immature barley embryo culture [38]. Other researchers have shown that light intensity and photoperiod exert a significant influence on shoot regeneration from a callus [36, 39]. However, there are few reports related to the specific molecular mechanism of the effect of light on the regeneration of embryo-derived EC in maize. In this study, we found that many specifically upregulated DEGs in the high-regeneration lines were related to photosynthesis. Furthermore, plant circadian rhythm was the most significant pathway enriched with downregulated DEGs in the low-regeneration lines. It is clear that photosynthesis and plant circadian rhythm both require light (Additional file 12: Figure S5 and Additional file 15: Figure S8). We speculated that light might regulate callus regeneration mainly by responding to photosynthesis and plant circadian rhythm.

Photosynthesis is the basic energy conversion process on Earth, facilitating the utilization of the energy from sunlight by living organisms [42]. In this study, photosynthesis, porphyrin and chlorophyll metabolism, and photosynthetic antenna proteins were the three significantly upregulated enriched pathways in the high-regeneration lines. Photosynthetic antenna proteins, which are specialized pigment–protein complexes, allow for the capture of energy from sunlight, thereby participating in the initial step of photosynthesis [43]. Chlorophyll, a compound of magnesium porphyrin that absorbs energy from light, is one of the most important pigments related to photosynthesis [44]. The DEGs involved in the pathway of photosynthesis were associated with photosystem I (PS I), photosystem II (PS II), photosynthetic electron transport, and F-type ATPase, all of which play significant roles in the process of photosynthesis. Sergio et al. showed that adventitious root formation in leafy cuttings of hazelnut (*Corylus avellana* L.) was affected by leaf photosynthesis, which provides the carbohydrate supply for this intensive metabolic process [45]. Some previous studies reported that the process of somatic embryogenesis and embryo germination demonstrated photosynthetic capacity [46–48]. The present study also indicated that EC gradually developed the photosynthetic apparatus and photosynthetic capacity for further autotrophy in this regeneration process, considering that the relative expression levels of most DEGs related to photosynthesis were gradually increased (Fig. 6a and Additional file 12: Figure S5).

Plant circadian rhythms are associated with the synchrony of the plant with the light cycle of its surrounding environment, providing the plant with information on the season, thereby informing flowering and pollinator attraction [49, 50]. In the pathway of plant circadian rhythm, Dof zinc finger proteins, phytochrome A and B (PHYA, PHYB), cryptochrome 1 (CRY1) were associated with downregulated expression in the low regeneration lines. Of these, Dof proteins, as transcription factors, are a subfamily of zinc finger proteins particular to the plant kingdom that are essential in the regulation of many plant growth and development processes, such as seed germination and the expression of some genes associated with photosynthesis [51, 52]. Moreover, PHYA is the main phytochrome in seedlings grown in the dark but rapidly degrades in light to produce CRY1 [49, 50]. Therefore, the downregulated expression of the DEGs related to plant circadian rhythms might be detrimental to the redifferentiation of EC (Fig. 6b and Additional file 15: Figure S8).

### Effects of plant hormones on callus regeneration

A number of DEGs were related to plant hormone signal transduction in both the high-and low-regeneration lines. Interestingly, in 141 and DH40, the specific common DEGs involved in hormone signal transduction were mainly associated with the signal transduction of JA, GA, BR, and ABA, whereas the majority of those in ZYDH381–1 and DH3732 were related to the signal transduction of auxin, CTK, BR, and GA (Additional file 16: Figure S9 and Additional file 17: Figure S10). JA plays an important role in plant growth and development, the stress response, and secondary metabolism processes, and JAZ is a plant-specific negative regulator of JA responsive genes, containing the conserved domain of ZIM and the Jas domain [53, 54]. In tissue culturing of the garlic bulb, the efficiency of shoot regeneration was improved by the addition of 1–10 μM JA in B5 basic medium [55]. In our study, JAZ was significantly downregulated in 141 and DH40. Given its negative regulation of downstream JA responsive genes, we speculated that the downregulated expression of these genes prompted the expression of downstream responsive genes and therefore motivated the regeneration of the callus. GA regulates plant growth by influencing stem growth and inducing germination [56], and the receptor protein GID1, DELLA protein, and PIF are involved in the signal transduction pathway of GA. In the tissue culture of immature barley embryo, the accumulation of ABA inhibited the shoot regeneration of the callus derived from immature barley embryos [57]. The downregulated DEGs related to the transduction of GA and ABA in our study might play a role in the regeneration of EC derived from immature maize embryos. Previous studies indicated that the balance of auxin and CTK is key to controlling the dedifferentiation and differentiation of plant cells, and either shoots or roots can be regenerated from a callus by adjusting the auxin–cytokinin ratios of the induction medium [58–60]. For example, a study on de novo shoot regeneration in *Arabidopsis thaliana* revealed that ARF3 directly bound to the promoter of *ATP/ADP ISOPENTENYLTRANSFERASE5* (*AtIPT5*) and negatively regulated *AtIPT5* expression, thereafter causing the abnormal biosynthesis of CTK and ultimately resulting in organ regeneration disruption [60]. Our study indicated that most of the DEGs related to auxin and CTK transduction were increased in 141 and DH40 but decreased in the poor regeneration lines (Fig. 6c), suggesting that the transduction of auxin and CTK is critical for callus regeneration. BR is a naturally produced class of plant steroid hormones and is typically involved in cell elongation, cell division, and differentiation throughout the plant life cycle and regulates many developmental processes, from seed germination to flowering and senescence [61, 62]. Research on cotton regeneration via somatic embryogenesis showed that BR had a stimulatory effect on the maturation of somatic embryos, but negatively affected callus growth [63, 64]. In this study, the DEGs associated with the transduction of BR were downregulated in 141 and DH40 but displayed inverse trends in the other two lines, which indicated that the transduction of BR might be negatively correlated with EC redifferentiation. In combination, these findings suggest that the regeneration process of embryogenic callus depends on the coordination of various hormones.

### Phenylpropanoid biosynthesis in callus regeneration

In our previous studies, the low-regenerating embryonic calli turned brown more easily, which is associated with cell disorganization and eventual cell death. Phenylpropanoid biosynthesis is induced by several stresses [65, 81], and we found it had significantly enriched pathways with specific common DEGs in ZYDH381–1 and DH3732, mainly including POD, PAL, and β-glucosidase. Notably, all of the DEGs were upregulated in ZYDH381–1 and DH3732 but downregulated or unchanged in 141 and DH40. Some studies have indicated that callus browning is a typical feature and a major obstacle in callus culturing and is related to polyphenol oxidase (PPO) and POD enzymatic activities [66–68]. A study on the plant regeneration of pine species via somatic organogenesis showed that cell death was correlated with elevated levels of peroxides, and the addition of antioxidants inhibited callus browning by reducing the accumulation of peroxidase [28]. PAL activity is correlated with xylogenesis and nodule induction in bean callus culture [69]. β-glucosidase activity is associated with cell lignification, and the production of lignin compounds in the callus of pine species was considered to be the result of stress reactions [70]. In the present study, the upregulated expression of genes related to phenylpropanoid biosynthesis was probably due to the response to environmental stress during EC regeneration, which might cause embryonic callus browning and hinder embryonic callus regeneration.

### The role of other regulators involved in callus regeneration

Some genes involved in cell cycle regulation and embryogenesis were differentially expressed, including FtsH, CDK, CCNA, CCNB, and WOX. Most of the cell cycle-related genes were upregulated in ZYDH381–1 and DH3732 but downregulated or unchanged in 141 and DH40, except embryogenesis-related genes (WOX), which were upregulated in 141 and DH40 but downregulated in ZYDH381–1 and DH3732. Among them, FtsH is the main thylakoid membrane protease found in organisms that performs oxygenic photosynthesis, and its malfunction causes cell division defects and growth arrest [35, 71]. Cell cycle regulation involves the differential expression of some cell-cycle genes during all phases of plant development. CDKs are a type of core cell cycle regulator that are regulated by the presence of cyclins [33, 72, 73]. We discovered that most of the cell cycle-related DEGs (FtsH, CDK, CCNA, CCNB) were significantly upregulated in the two poor regeneration materials but exhibited opposite trends in the two high-regeneration materials. Accordingly, we suggested that the increased expression of these DEGs facilitated the maintenance of the condition of EC and hindered the redifferentiation of EC. The WOX family (with 15 members) is a class of transcription factors that specifically exists in plants, including WUS and WOX1-WOX14 in *Arabidopsis*, and plays an important role in the stem cell maintenance of SAM and RAM, the development of lateral organs, the formation of floral organs, and embryo development [74–77]. Previous studies showed that WOX played an ancestral role in embryo development in seed plants, and the connection among polar auxin transport (PAT), PIN-FORMED (PIN), and WOX in the regulation of embryo patterning in seed plants was strengthened by the study of Palovaara et al. [78]. Lowe et al. also obtained plantlets with increased resistance from transgenic callus by overexpressing the maize *BBM* and *WUSCHEL2 (WUS2)* genes [79]. In this study, the *WOX* genes were upregulated in the two high-regeneration capacity materials but downregulated in the other lines, which verified the positive regulatory role of *WOX* genes in callus regeneration.

## Conclusions

In maize, the obvious differences in induction and regeneration capabilities of EC among various genotypes result in genotype dependence in genetic transformation. In this study, transcriptome analysis of the EC of the four maize inbred lines showed that the specific common DEGs of the high-regeneration lines were mainly associated with photosynthesis, porphyrin and chlorophyll metabolism, ribosomes, and plant hormone signal transduction, while those of the low regeneration lines were mainly related to taurine and hypotaurine metabolism, nitrogen metabolism, fatty acid elongation, starch and sucrose metabolism, phenylpropanoid biosynthesis, and plant circadian rhythm. More importantly, *WOX* genes that have an ancestral role in embryo development in seed plants and promote the regeneration of transformed calli were specifically upregulated in the two high-regeneration lines. Our research provides new insight into molecular regulation during the early redifferentiation of a maize embryonic callus.

## Methods

### Samples and RNA isolation

In our previous study, we obtained two high-regeneration lines (141, DH40) and two low-regeneration lines (ZYDH381–1 and DH3732) from a natural population of 144 maize inbred lines [85, 89]. Furthermore, 141 and DH3732 both belong to NSS group, whereas DH40 and ZYDH381–1 were classified into Tropical group and SS group, respectively [90]. In the present study, the immature embryos of 1.0–1.5 mm in length were collected from the four lines at 12 d after self-pollination and then cultured in modified N6 medium supplemented with 2,4-D under aseptic and aphotic conditions at 27 °C for 25 d to induce embryonic callus (EC). The embryo-derived EC was subsequently transferred to new N6 medium and cultured for 20 d to subculture the EC, which was then cultured in MS medium at 27 °C in 12 h light/d for 9 d for redifferentiation. No replacement of fresh media was conducted during each of the culture phases. The specific components of the medium were listed in Additional file 20: Table S1. Total RNA was isolated from a pool of three calli for each sample at 0–9 d during redifferentiation by using Trizol Reagent (Invitrogen). RNA from 1 to 3 d samples after redifferentiation (stage I sample, green callus forming) were mixed in equal proportions, as were RNA from 4 to 6 d (stage II sample, embryoid and less adventitious bud emerged) and 7–9 d (stage III sample, mass adventitious bud forming), with the 0 d RNA being used as the control (CK). The four RNA samples of each line were then separately submitted to transcriptome analysis using Illumina sequencing, with three biological replicates.

### Illumina sequencing and data analysis

Clean reads were obtained by filtering low-quality reads, adaptor-polluted reads, and reads with a high content of unknown base (N) reads. Then, the clean reads were mapped to the reference genome and genes of maize available at RefGen_V4 ( http://www.gramene.org/) using Bowtie2. Gene expression levels were calculated with RSEM. The Fragments Per Kilobase of transcript per Million mapped reads (FPKM) method was used to estimate transcript expression levels in all the samples. DESeq2 was used to detect differentially expressed genes (DEGs) between each selected sample pair. The DESeq2 parameters included fold change (FC) ≥ 2.00 and adjusted *P*-value ≤0.05. Hierarchical clustering of the DEGs was performed using the pheatmap package in R. The DEGs were clustered according to their expression levels using ExpressCluster software to investigate their expression patterns. Gene Ontology (GO) and pathway annotations and enrichment analyses were based on the Gene Ontology Database (www.geneontology.org) [21] and Kyoto Encyclopedia of Genes and Genomes (KEGG) pathway (www.genome.jp/kegg) [22], respectively. The calculated *P*-values was subjected to Bonferroni correction [23], using the corrected *P*-value ≤0.05 as a threshold.

### DEG statistics

**Fig. 7:**
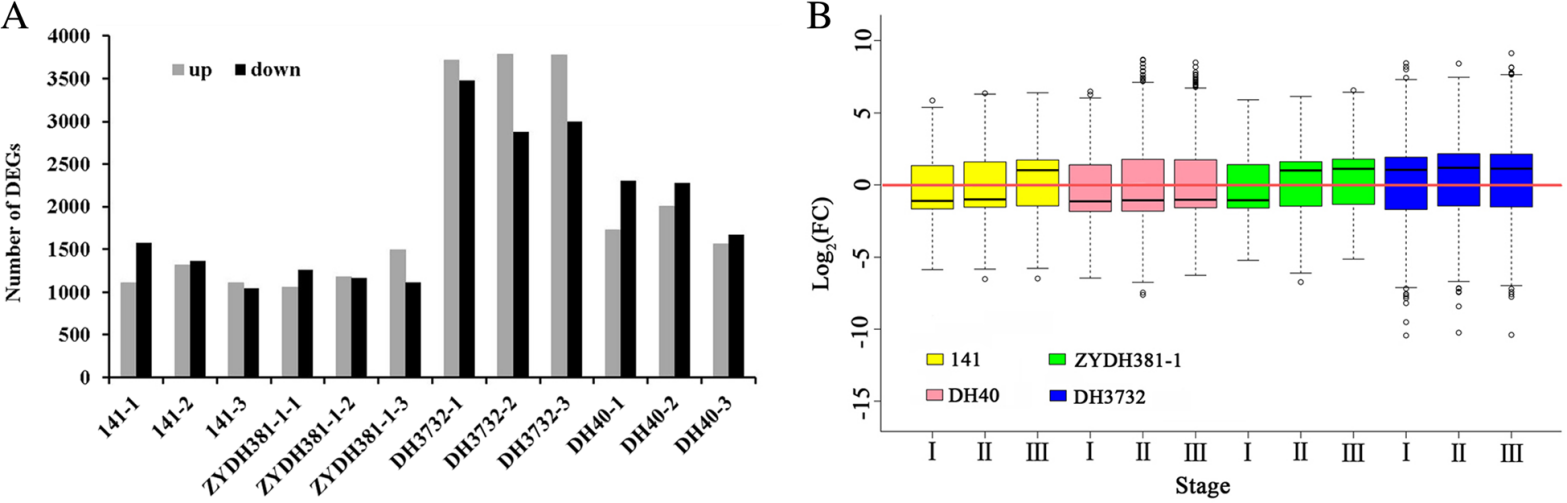
Numbers and transcription levels of DEGs of the four maize inbred lines. **a** Number of DEGs for each maize. inbred line. **b** Relative expression levels of the DEGs at the three stages of EC redifferentiation for each maize inbred line. Each box plot shows the distribution of the relative transcription level [log2 (fold-change)] of the DEGs. The red line indicates a one-fold change relative to the transcription level of the control samples

In this study, DESeq2 was used to identify the DEGs between each comparison with a threshold of false discovery rate (FDR)-adjusted *P-*value ≤0.05 and FC ≥ 2 (|Log_2_FC| ≥ 1) [22]. A large number of DEGs were detected at the three stages of EC regeneration for each inbred line in comparison to the control (Fig. 7a). At stage I, there were 1116, 1727, 1059, and 3714 upregulated DEGs and 1578, 2304, 1265, and 3479 downregulated DEGs for lines 141, DH40, ZYDH381–1, and DH3732, respectively. For Stage II, there were 1322, 2007, 1182, and 3784 upregulated DEGs and 1367, 2277, 1171, and 2876 downregulated DEGs in 141, DH40, ZYDH381–1, and DH3732, respectively. In contrast, in Stage III, 1108, 1563, 1498, and 3779 DEGs showed increased expression for 141, DH40, ZYDH381–1, and DH3732, respectively, and 1043, 1668, 1112, and 2999 displayed decreased expression.

The expression level ranges of the DEGs are displayed in Fig. 7b. In line 141, the relative expressions (compared to CK) of these DEGs ranged from − 5.864 to 5.863, − 6.516 to 6.366, and − 6.479 to 6.404 at stage I, stage II, and stage III, respectively. For DH40, the FC of the DEGs ranged from − 6.449 to 6.488, − 7.613 to 8.679, and − 6.257 to 8.477 in the three stages, respectively. The expression of the DEGs in ZYDH381–1 ranged from − 5.214 to 5.914, − 6.726 to 6.144, and − 5.130 to 6.573 at stage I, stage II, and stage III, respectively. In DH3732, these DEGs were more sensitive to EC regeneration than the other lines, with FC values ranging from − 10.440 to 8.431, − 10.246 to 8.402, and − 10.397 to 9.122 at these stages, respectively.

### Quantitative real-time PCR validation

To validate the DEGs obtained from the Illumina sequencing, 10 genes (*Zm00001d041327*, *Zm00001d033049*, *Zm00001d018178*, *Zm00001d047789*, *Zm00001d019518*, *Zm00001d018157*, *Zm00001d008230*, *Zm00001d022041*, *Zm00001d049387*, *Zm00001d014723*) were randomly selected to conduct quantitative real-time PCR analysis using an Applied Biosystems 7500 Real-Time PCR System. *GADPH* (*Zm00001d049641*) was used as the endogenous control. cDNA synthesis was carried out using 1 μg of total RNA. The corresponding primers were designed using the Primer 3 online tool and are listed in Additional file 21: Table S2. The amplification program was performed according to the standard protocol of the Applied Biosystems 7500 Real-Time PCR System: 40 cycles of 98 °C for 2 min, 98 °C for 2 s, and 59 °C for 10 s, followed by a thermal denaturing step to generate the melting curves for amplification specificity verification. All reactions were run in triplicate, including non-template controls. Statistical analysis was performed using the 2^-△△CT^ method [87].

## Additional files

Additional file 1: **Figure S1.** Base composition and quality of clean data. (JPG 1466 kb)

Additional file 2: **Figure S2.** CorrelationHeatmap of AllSamples. (PDF 17 kb)

Additional file 3: **Table S3.** The detailed information about the specific common DEGs of 141 and DH40; **Table S4.** The detailed information about the specific common DEGs of DH3732 and ZYDH381–1.). (XLSX 874 kb)

Additional file 4: **Table S5.** Up specific common DEGs of the two high regeneration lines in 5 clusters; **Table S6.** Down specific common DEGs of the two high regeneration lines in 5 clusters; **Table S7.** Up specific common DEGs of the two low regeneration lines in 5 clusters; **Table S8.** Down specific common DEGs of the two low regeneration lines in 5 clusters.). (XLSX 195 kb)

Additional file 5: **Figure S3.** GO analysis of specific common DEGs of 141 and DH40 (A. up-regulated gene; B. down-regulated gene) (JPG 1422 kb)

Additional file 6: **Table S9.** List of GO analysis (BP) for the specific common DEGs of 141 and DH40 (All GO terms shown were significant at FDR ≤ 0.05); **Table S10.** List of GO analysis (CC) for the specific common DEGs of 141 and DH40 (GO terms shown were significant at FDR ≤ 0.05 for up-regulated genes, and *P*-value ≤0.05 for down-regulated genes); **Table S11.** List of GO analysis (MF) for the specific common DEGs of 141 and DH40 (All GO terms shown were significant at FDR ≤ 0.05).). (DOCX 45 kb)

Additional file 7: **Figure S4.** GO analysis of specific common DEGs of ZYDH381–1 and DH3732 (A. up-regulated gene; B. down-regulated gene) (JPG 2215 kb)

Additional file 8: **Table S12.** List of GO analysis (BP) for the specific common DEGs of DH3732 and ZYDH381–1 (All GO terms shown were significant at FDR ≤ 0.05); **Table S13.** List of GO analysis (CC) for the specific common DEGs of DH3732 and ZYDH381–1 (All GO terms shown were significant at FDR ≤ 0.05); **Table S14.** List of GO analysis (MF) for the specific common DEGs of DH3732 and ZYDH381–1 (All GO terms shown were significant at FDR ≤ 0.05).). (DOCX 20 kb)

Additional file 9: **Table S15.** List of enriched pathways for the specific common DEGs of 141 and DH40 at three stages (stage I II and III). (XLSX 32 kb)

Additional file 10: **Table S16.** List of enriched pathways for specific common DEGs of ZYDH381–1 and DH3732 at three stages (stage I II and III). (XLSX 44 kb)

Additional file 11: **Table S17.** List of DEGs related to embryonic callus regeneration. (DOCX 20 kb)

Additional file 12: **Figure S5.** KEGG Pathway Map of Photosynthesis for the specific common DEGs of 141 and DH40. (PNG 43 kb)

Additional file 13: **Figure S6.** KEGG Pathway Map of Protein and Chlorophyll Metabolism. (PNG 42 kb)

Additional file 14: **Figure S7.** KEGG Pathway Map of Photosynthesis-Antenna Protein. (PNG 50 kb)

Additional file 15: **Figure S8.** KEGG Pathway Map of Circadian Rhythm-Plant. (PNG 13 kb)

Additional file 16: **Figure S9.** KEGG Pathway Map of Plant Hormone Signal Transduction for the specific common DEGs of 141 and DH40. (PNG 445 kb)

Additional file 17: **Figure S10.** KEGG Pathway Map of Plant Hormone Signal Transduction for the specific common DEGs of ZYDH381–1 and DH3732. (PNG 152 kb)

Additional file 18: **Figure S11.** KEGG Pathway Map of Phenylpropanoid Biosynthesis. (PNG 21 kb)

Additional file 19: **Figure S12.** Expression clustering of specific common DEGs involved in EC regeneration. (JPG 4769 kb)

Additional file 20: **Table S1.** Plant culture medium formula. (DOCX 14 kb)

Additional file 21: **Table S2.** Primers of real-time qRT-PCR assay used in this study. (DOCX 15 kb)

## Abbreviations

ABA: Abscisic acid
ARF: Auxin response factor
BP: Biological Process
BR: Brassinosteroid
BRI: Protein brassinosteroid insensitive 1
CC: Cellular Component
CCNA: Cyclin A
CCNB: Cyclin B
CDK: Cyclin-dependent kinase
CDR: Callus differentiating rate
CK: Control
CPN: Callus plantlet number
CTK: Cytokinin
DEGs: Differentially expressed genes
EBF: EIN3-binding F-box protein
EC: Embryonic callus
EIN3: Ethylene-insensitive protein 3
FPKM: Million mapped reads
FtsH: Cell division protease FtsH
GA: Gibberellin
GID1: Gibberellin receptor GID1
GO: Gene Ontology
JA: Jasmonic acid
KEGG: Kyoto Encyclopedia of Genes and Genomes
MF: Molecular Function
MYC2: Transcription factor MYC2
PAL: Phenylalanine ammonia-lyase
PIF4: Phytochrome-interacting factor 4
POD: Peroxidase
RAMs: Root apical meristems
SAM: Shoot apical meristem
SAUR: SAUR family protein
WOX: WUSCHEL homeobox protein
WUS: WUSCHEL
