# Supplementary figures and images for: Transcriptome sequencing analysis of maize embryonic callus during early redifferentiation
Source: BMC Genomics. 2019 Feb 27;20:159. doi: 10.1186/s12864-019-5506-7 (PMC6391841; doi:10.1186/s12864-019-5506-7)

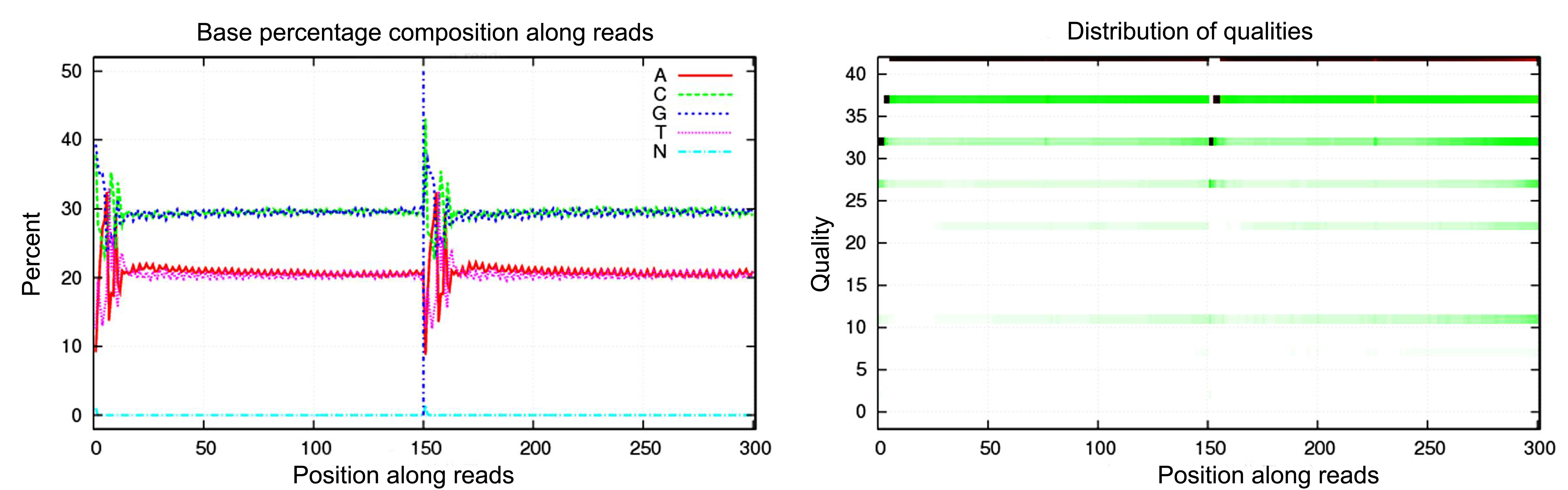

Supplement: Supplementary file 1 — Figure S1. Base composition and quality of clean data. (JPG 1466 kb) [file 12864_2019_5506_MOESM1_ESM.jpg]

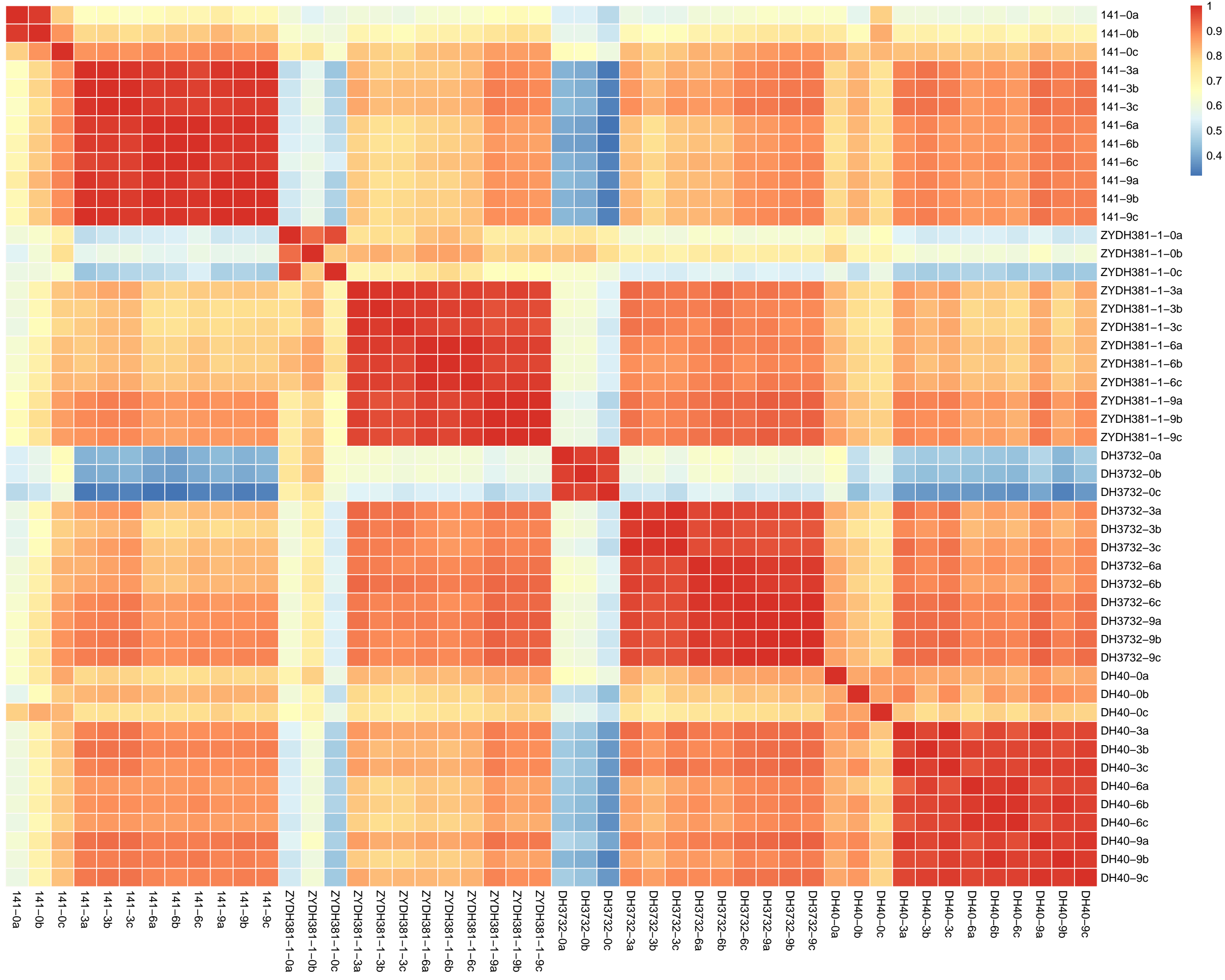

Supplement: Supplementary file 2 — Figure S2. CorrelationHeatmap of AllSamples. (PDF 17 kb) [file 12864_2019_5506_MOESM2_ESM.pdf]

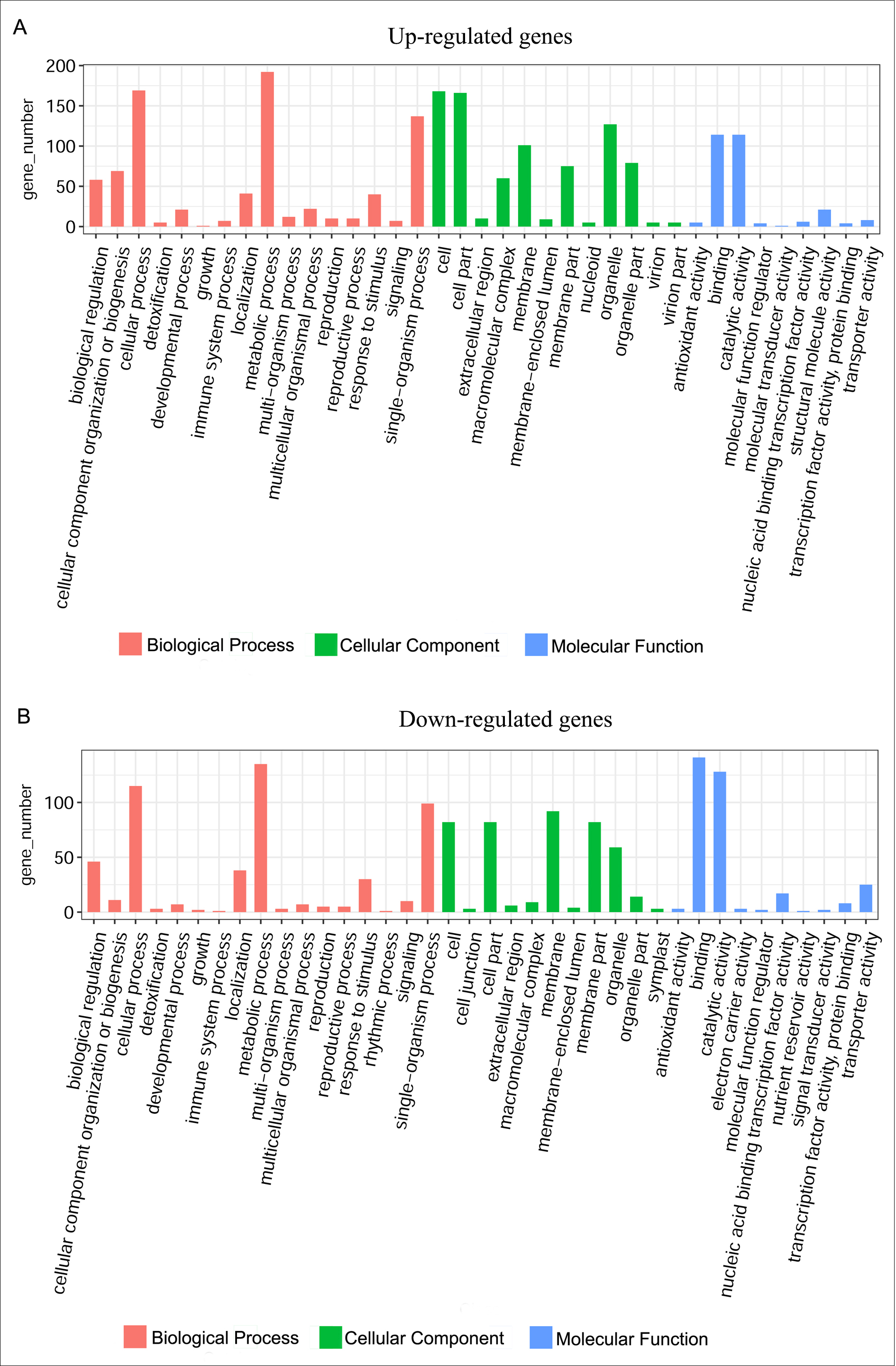

Supplement: Supplementary file 5 — Figure S3. GO analysis of specific common DEGs of 141 and DH40 (A. up-regulated gene; B. down-regulated gene) (JPG 1422 kb) [file 12864_2019_5506_MOESM5_ESM.jpg]

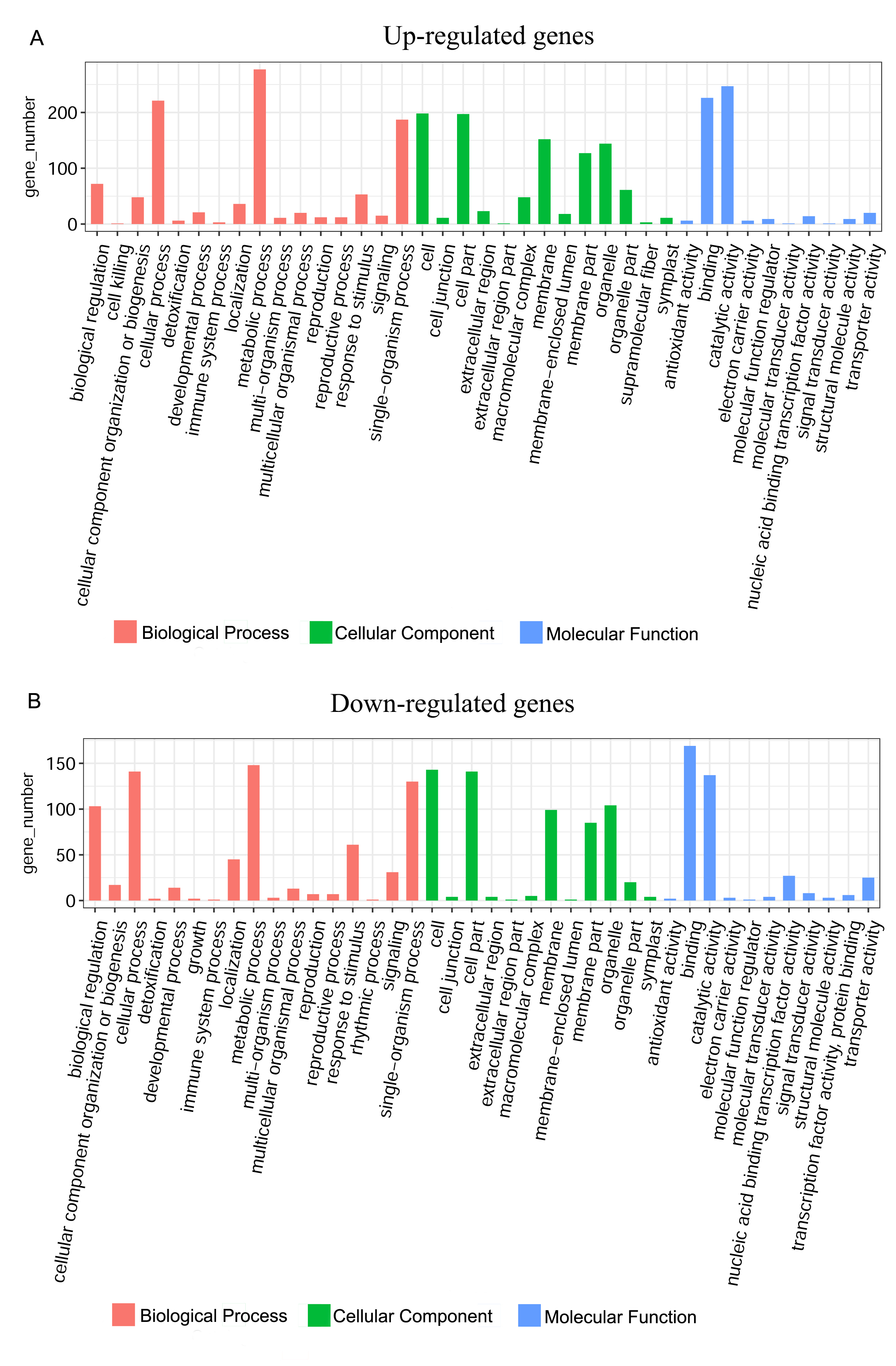

Supplement: Supplementary file 7 — Figure S4. GO analysis of specific common DEGs of ZYDH381–1 and DH3732 (A. up-regulated gene; B. down-regulated gene) (JPG 2215 kb) [file 12864_2019_5506_MOESM7_ESM.jpg]

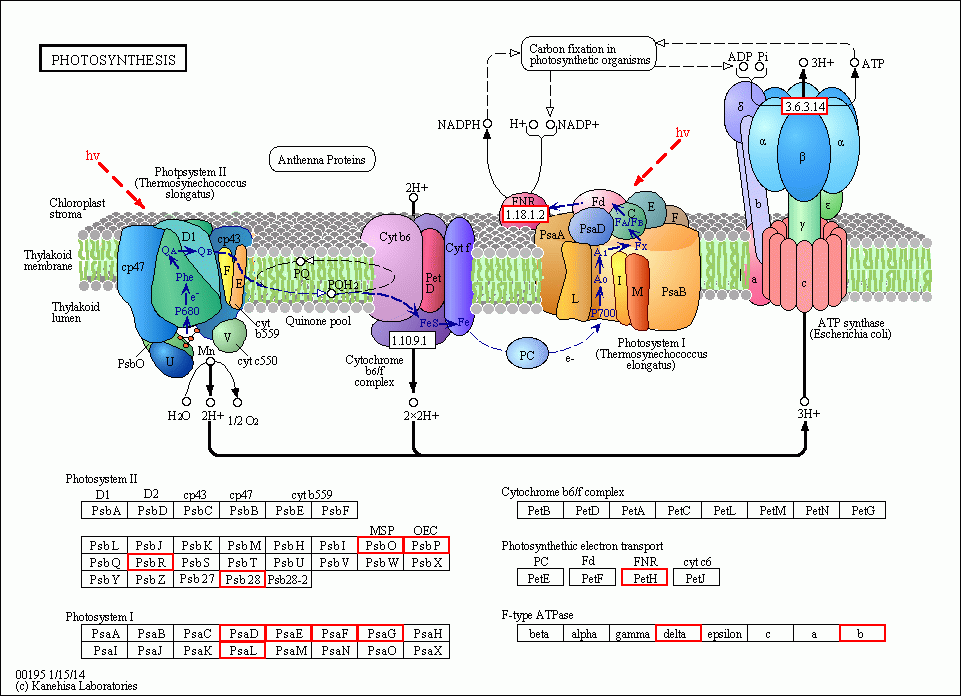

Supplement: Supplementary file 12 — Figure S5. KEGG Pathway Map of Photosynthesis for the specific common DEGs of 141 and DH40. (PNG 43 kb) [file 12864_2019_5506_MOESM12_ESM.png]

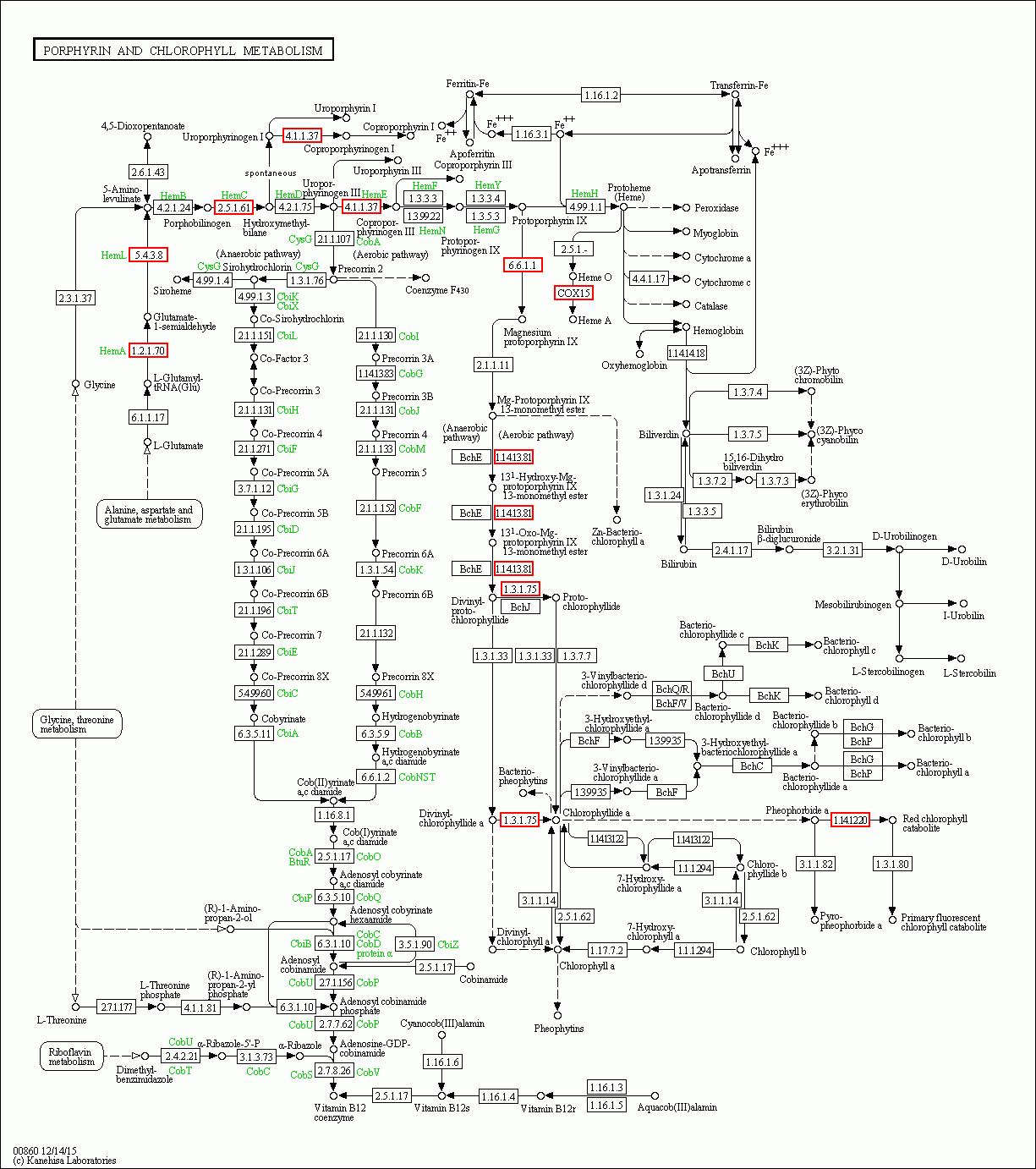

Supplement: Supplementary file 13 — Figure S6. KEGG Pathway Map of Protein and Chlorophyll Metabolism. (PNG 42 kb) [file 12864_2019_5506_MOESM13_ESM.png]

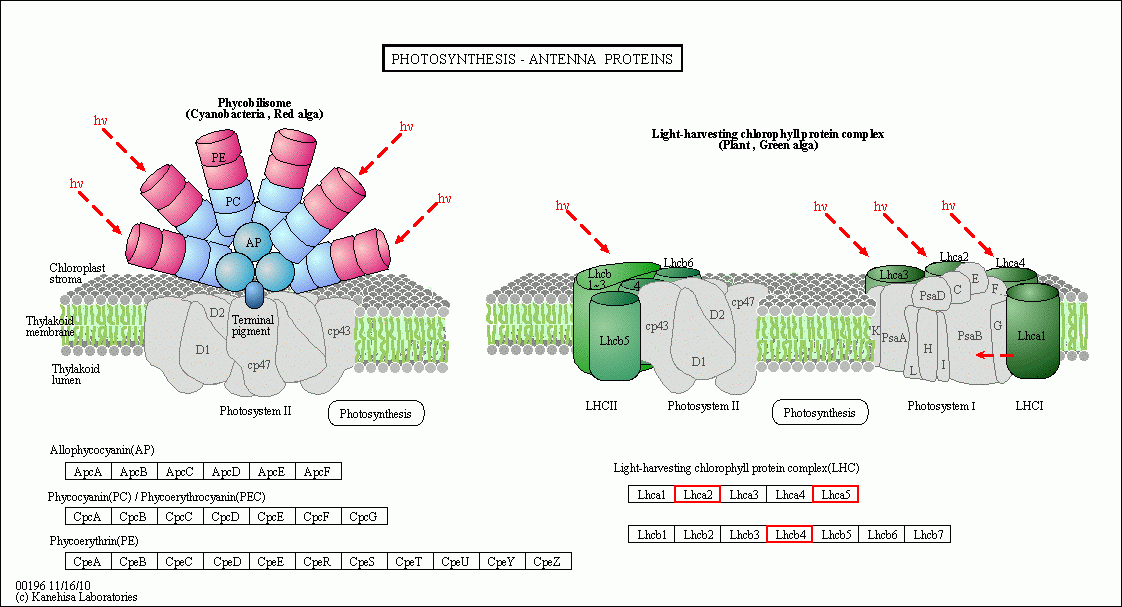

Supplement: Supplementary file 14 — Figure S7. KEGG Pathway Map of Photosynthesis-Antenna Protein. (PNG 50 kb) [file 12864_2019_5506_MOESM14_ESM.png]

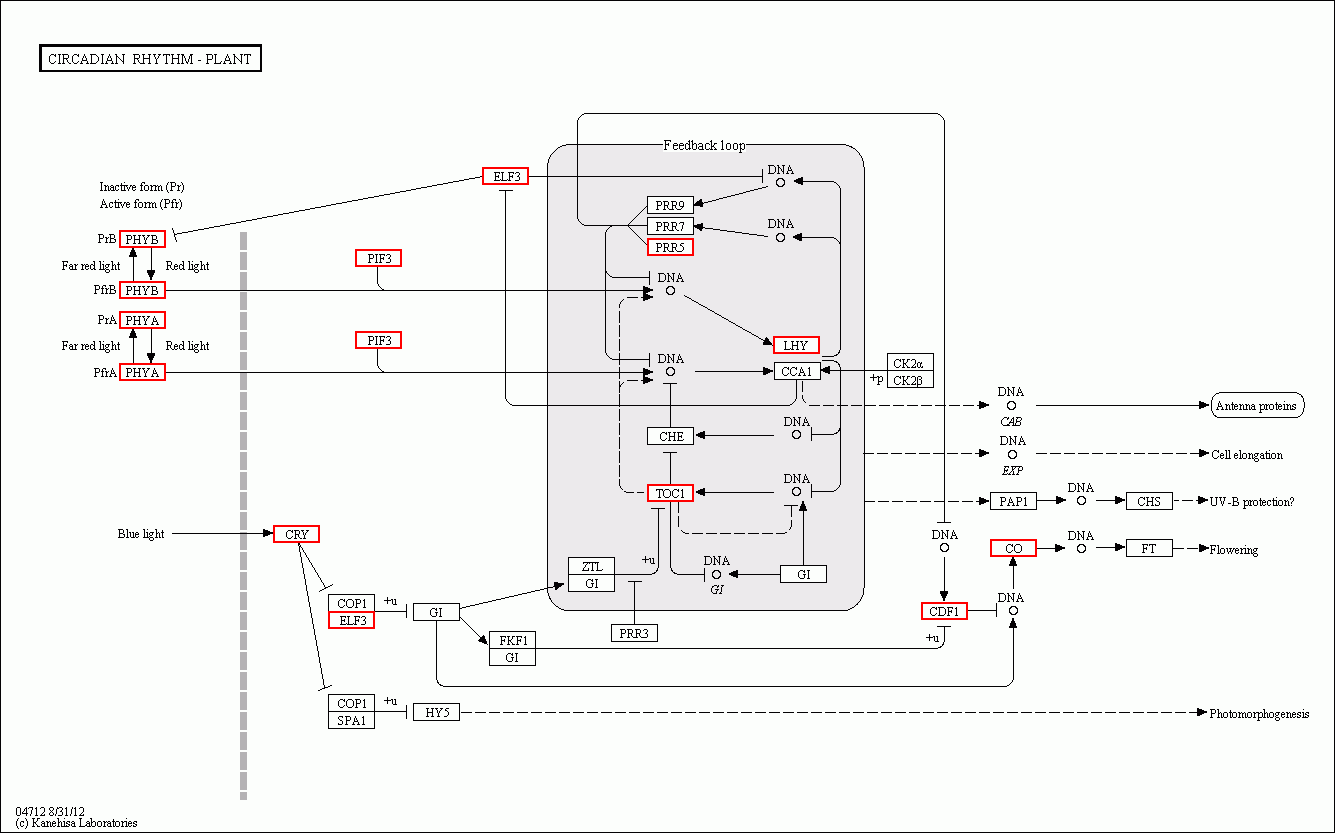

Supplement: Supplementary file 15 — Figure S8. KEGG Pathway Map of Circadian Rhythm-Plant. (PNG 13 kb) [file 12864_2019_5506_MOESM15_ESM.png]

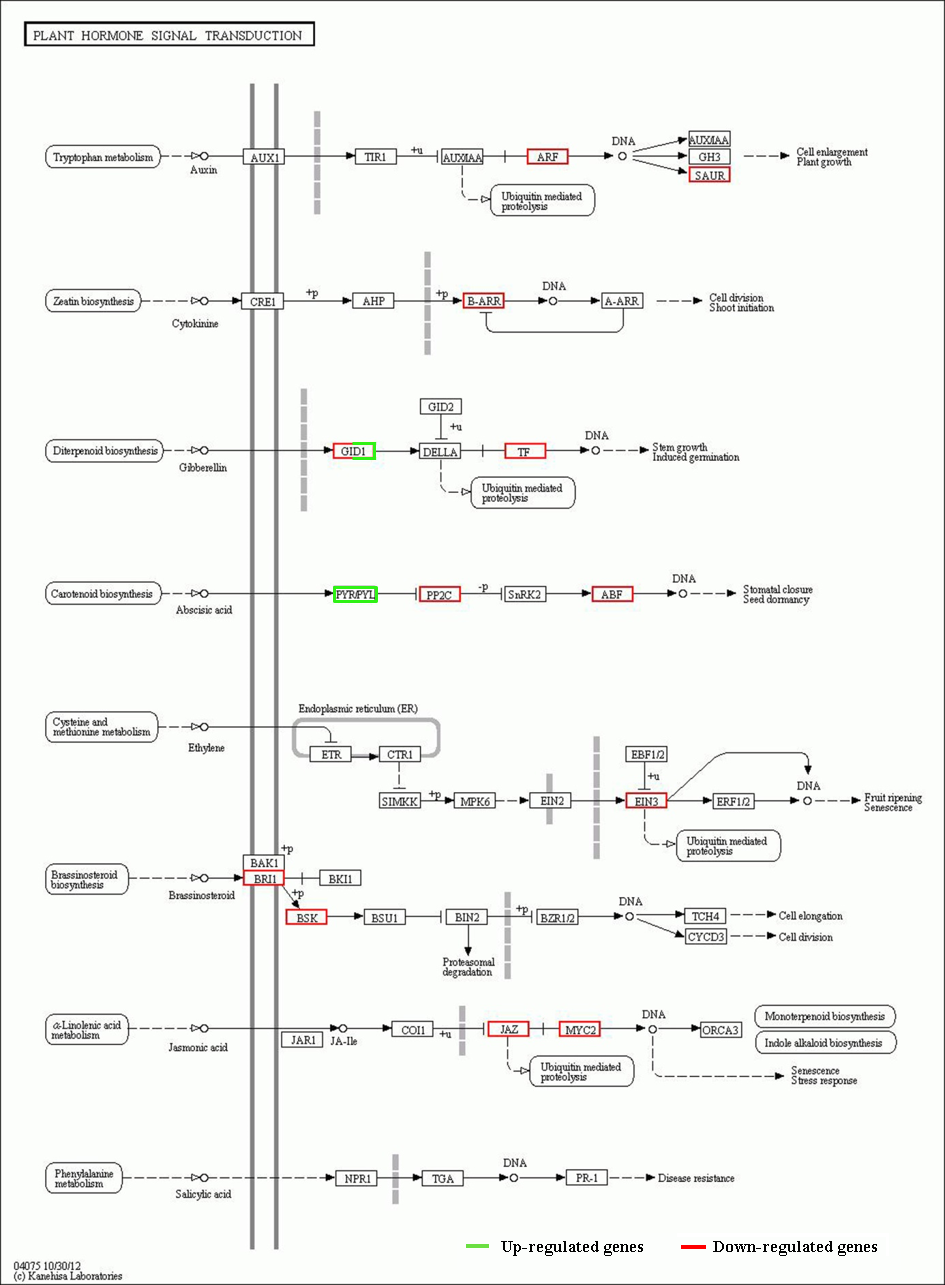

Supplement: Supplementary file 16 — Figure S9. KEGG Pathway Map of Plant Hormone Signal Transduction for the specific common DEGs of 141 and DH40. (PNG 445 kb) [file 12864_2019_5506_MOESM16_ESM.png]

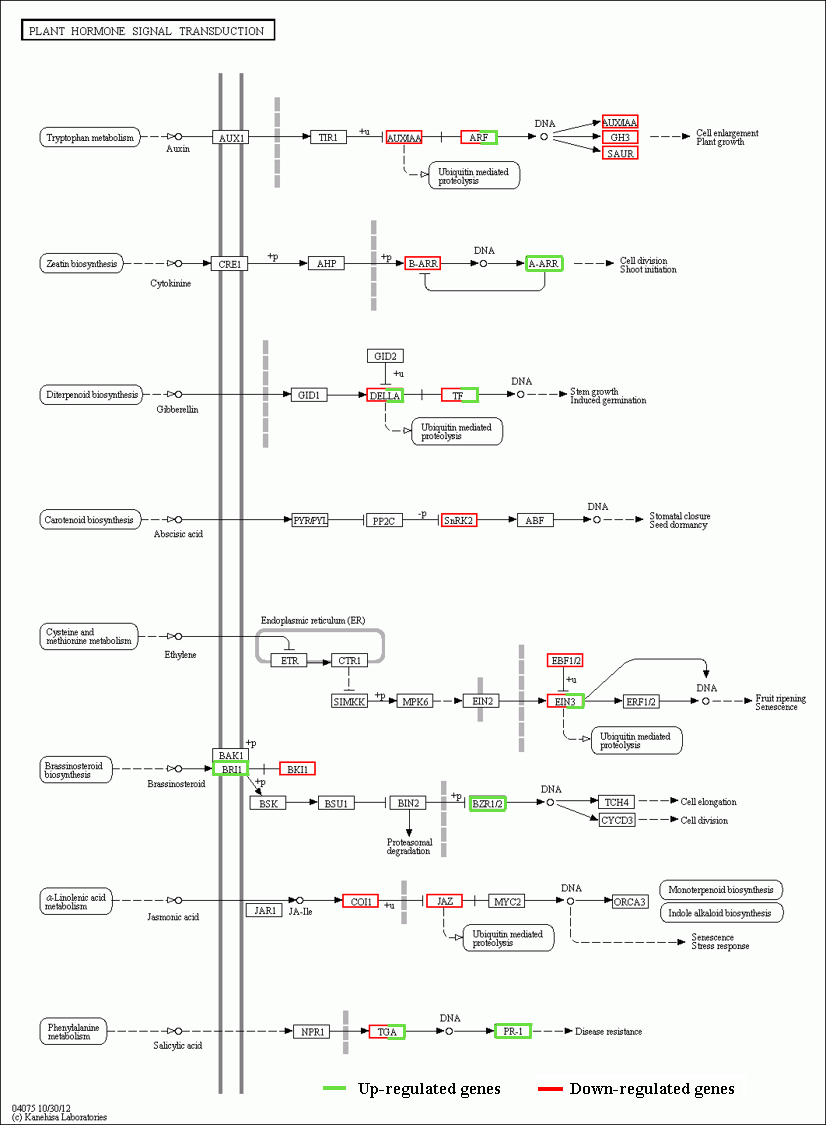

Supplement: Supplementary file 17 — Figure S10. KEGG Pathway Map of Plant Hormone Signal Transduction for the specific common DEGs of ZYDH381–1 and DH3732. (PNG 152 kb) [file 12864_2019_5506_MOESM17_ESM.png]

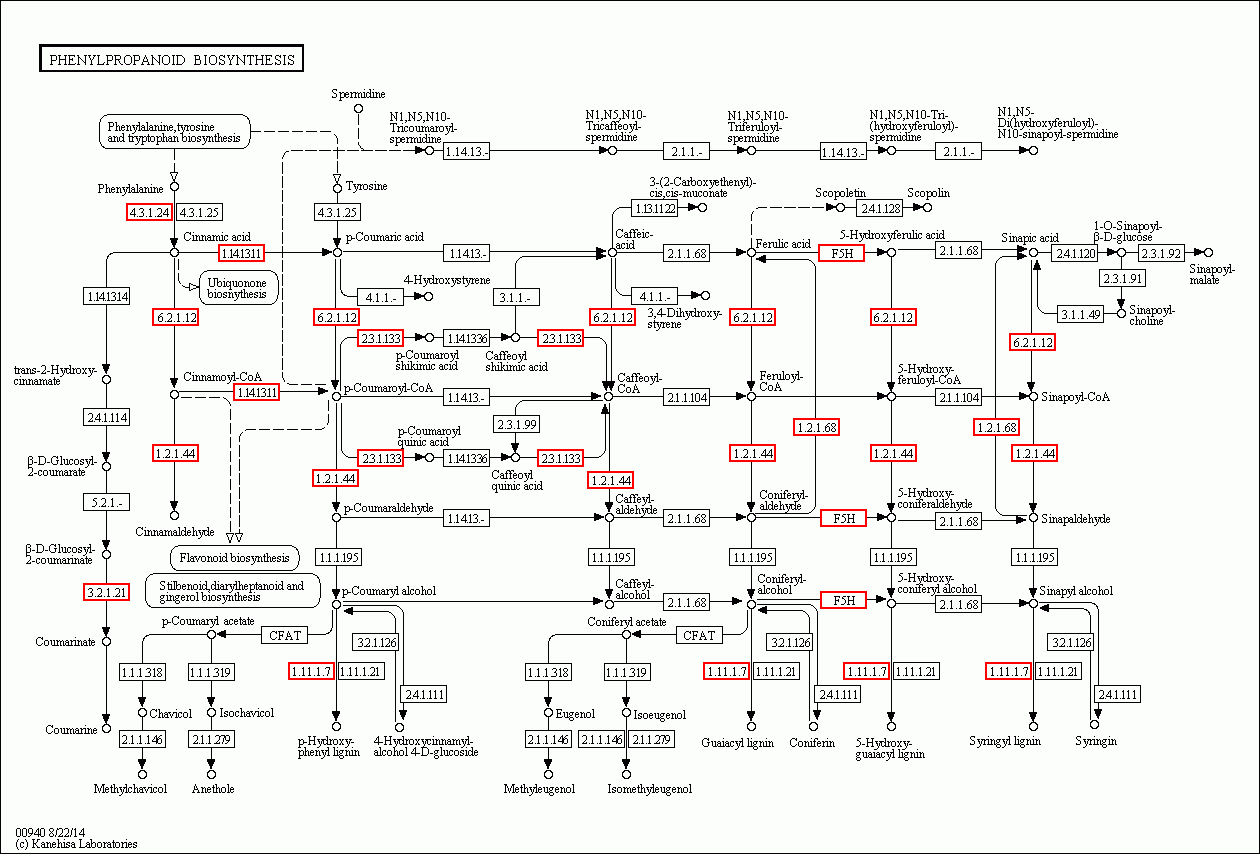

Supplement: Supplementary file 18 — Figure S11. KEGG Pathway Map of Phenylpropanoid Biosynthesis. (PNG 21 kb) [file 12864_2019_5506_MOESM18_ESM.png]

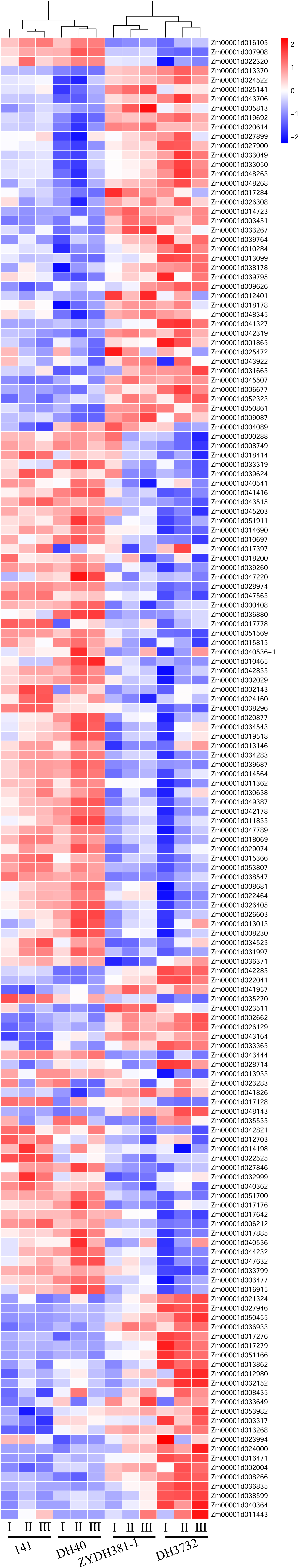

Supplement: Supplementary file 19 — Figure S12. Expression clustering of specific common DEGs involved in EC regeneration. (JPG 4769 kb) [file 12864_2019_5506_MOESM19_ESM.jpg]
