# Supplementary material for: Transcriptome sequencing analysis of maize embryonic callus during early redifferentiation
Source: BMC Genomics. 2019 Feb 27;20:159. doi: 10.1186/s12864-019-5506-7 (PMC6391841; doi:10.1186/s12864-019-5506-7)
Supplement: Supplementary file 6 — Table S9. List of GO analysis (BP) for the specific common DEGs of 141 and DH40 (All GO terms shown were significant at FDR ≤ 0.05); Table S10. List of GO analysis (CC) for the specific common DEGs of 141 and DH40 (GO terms shown were significant at FDR ≤ 0.05 for up-regulated genes, and P-value ≤0.05 for down-regulated genes); Table S11. List of GO analysis (MF) for the specific common DEGs of 141 and DH40 (All GO terms shown were significant at FDR ≤ 0.05).). (DOCX 45 kb) [file 12864_2019_5506_MOESM6_ESM.docx]

Table S9 List of GO analysis (**BP**) for the specific common DEGs of 141 and DH40 (All GO terms shown were significant at FDR ≤ 0.05).

| **GO ID** | **GO Term** | **P-value** | **FDR** |
| --- | --- | --- | --- |
| **Up-regulated genes** | |  |  |
| GO:0015979 | photosynthesis | 1.10E-50 | 1.04E-47 |
| GO:0019684 | photosynthesis, light reaction | 2.99E-37 | 1.43E-34 |
| GO:0019682 | glyceraldehyde-3-phosphate metabolic process | 3.26E-31 | 1.04E-28 |
| GO:0010207 | photosystem II assembly | 1.05E-27 | 2.51E-25 |
| GO:0019288 | isopentenyl diphosphate biosynthetic process, methylerythritol 4-phosphate pathway | 3.82E-27 | 7.28E-25 |
| GO:0009240 | isopentenyl diphosphate biosynthetic process | 5.51E-27 | 7.50E-25 |
| GO:0046490 | isopentenyl diphosphate metabolic process | 5.51E-27 | 7.50E-25 |
| GO:0006081 | cellular aldehyde metabolic process | 7.06E-27 | 8.41E-25 |
| GO:0009657 | plastid organization | 8.06E-27 | 8.54E-25 |
| GO:0008299 | isoprenoid biosynthetic process | 3.97E-24 | 3.79E-22 |
| GO:0006720 | isoprenoid metabolic process | 1.56E-23 | 1.35E-21 |
| GO:0006091 | generation of precursor metabolites and energy | 5.63E-22 | 4.47E-20 |
| GO:0008654 | phospholipid biosynthetic process | 4.26E-21 | 3.12E-19 |
| GO:0009668 | plastid membrane organization | 9.82E-21 | 6.24E-19 |
| GO:0010027 | thylakoid membrane organization | 9.82E-21 | 6.24E-19 |
| GO:0044711 | single-organism biosynthetic process | 2.48E-20 | 1.48E-18 |
| GO:0046148 | pigment biosynthetic process | 3.11E-20 | 1.74E-18 |
| GO:0042440 | pigment metabolic process | 2.36E-19 | 1.25E-17 |
| GO:0006090 | pyruvate metabolic process | 7.30E-19 | 3.66E-17 |
| GO:0033014 | tetrapyrrole biosynthetic process | 1.19E-18 | 5.68E-17 |
| GO:0033013 | tetrapyrrole metabolic process | 4.71E-18 | 2.14E-16 |
| GO:0006644 | phospholipid metabolic process | 6.04E-18 | 2.54E-16 |
| GO:1901566 | organonitrogen compound biosynthetic process | 6.13E-18 | 2.54E-16 |
| GO:0051186 | cofactor metabolic process | 9.52E-18 | 3.78E-16 |
| GO:0044249 | cellular biosynthetic process | 1.24E-17 | 4.69E-16 |
| GO:0043623 | cellular protein complex assembly | 1.28E-17 | 4.69E-16 |
| GO:0006364 | rRNA processing | 9.76E-17 | 3.45E-15 |
| GO:0044802 | single-organism membrane organization | 1.02E-16 | 3.48E-15 |
| GO:0016072 | rRNA metabolic process | 1.06E-16 | 3.48E-15 |
| GO:0008610 | lipid biosynthetic process | 1.48E-16 | 4.71E-15 |
| GO:1901564 | organonitrogen compound metabolic process | 2.77E-16 | 8.52E-15 |
| GO:1901576 | organic substance biosynthetic process | 3.41E-16 | 1.02E-14 |
| GO:0015995 | chlorophyll biosynthetic process | 9.02E-16 | 2.60E-14 |
| GO:0051188 | cofactor biosynthetic process | 1.00E-15 | 2.81E-14 |
| GO:0034660 | ncRNA metabolic process | 1.32E-15 | 3.59E-14 |
| GO:0009058 | biosynthetic process | 1.45E-15 | 3.83E-14 |
| GO:0044255 | cellular lipid metabolic process | 1.52E-15 | 3.91E-14 |
| GO:0006779 | porphyrin-containing compound biosynthetic process | 1.80E-15 | 4.53E-14 |
| GO:0061024 | membrane organization | 1.94E-15 | 4.74E-14 |
| GO:0006461 | protein complex assembly | 2.70E-15 | 6.42E-14 |
| GO:0070271 | protein complex biogenesis | 2.86E-15 | 6.65E-14 |
| GO:0015994 | chlorophyll metabolic process | 4.04E-15 | 9.16E-14 |
| GO:0006778 | porphyrin-containing compound metabolic process | 5.12E-15 | 1.13E-13 |
| GO:0044271 | cellular nitrogen compound biosynthetic process | 5.23E-15 | 1.13E-13 |
| GO:0043436 | oxoacid metabolic process | 5.36E-14 | 1.13E-12 |
| GO:0006082 | organic acid metabolic process | 5.50E-14 | 1.14E-12 |
| GO:0034470 | ncRNA processing | 9.15E-14 | 1.85E-12 |
| GO:0071822 | protein complex subunit organization | 1.01E-13 | 2.00E-12 |
| GO:0006739 | NADP metabolic process | 1.04E-13 | 2.02E-12 |
| GO:1901135 | carbohydrate derivative metabolic process | 1.64E-13 | 3.13E-12 |
| GO:0042254 | ribosome biogenesis | 1.74E-13 | 3.24E-12 |
| GO:0019752 | carboxylic acid metabolic process | 2.43E-13 | 4.45E-12 |
| GO:0044281 | small molecule metabolic process | 2.89E-13 | 5.19E-12 |
| GO:0044085 | cellular component biogenesis | 4.86E-13 | 8.55E-12 |
| GO:0032787 | monocarboxylic acid metabolic process | 4.93E-13 | 8.55E-12 |
| GO:0006629 | lipid metabolic process | 5.40E-13 | 9.19E-12 |
| GO:0006098 | pentose-phosphate shunt | 5.65E-13 | 9.44E-12 |
| GO:0090407 | organophosphate biosynthetic process | 7.02E-13 | 1.15E-11 |
| GO:0006807 | nitrogen compound metabolic process | 8.05E-13 | 1.30E-11 |
| GO:0022607 | cellular component assembly | 9.39E-13 | 1.49E-11 |
| GO:0034622 | cellular macromolecular complex assembly | 1.15E-12 | 1.80E-11 |
| GO:0051156 | glucose 6-phosphate metabolic process | 1.18E-12 | 1.82E-11 |
| GO:0019637 | organophosphate metabolic process | 1.63E-12 | 2.46E-11 |
| GO:0009902 | chloroplast relocation | 3.87E-12 | 5.50E-11 |
| GO:0019750 | chloroplast localization | 3.87E-12 | 5.50E-11 |
| GO:0051644 | plastid localization | 3.87E-12 | 5.50E-11 |
| GO:0051667 | establishment of plastid localization | 3.87E-12 | 5.50E-11 |
| GO:0051656 | establishment of organelle localization | 1.45E-11 | 2.04E-10 |
| GO:0022613 | ribonucleoprotein complex biogenesis | 1.50E-11 | 2.05E-10 |
| GO:0019438 | aromatic compound biosynthetic process | 1.51E-11 | 2.05E-10 |
| GO:0010467 | gene expression | 1.53E-11 | 2.05E-10 |
| GO:0009773 | photosynthetic electron transport in photosystem I | 1.69E-11 | 2.24E-10 |
| GO:0065003 | macromolecular complex assembly | 2.04E-11 | 2.66E-10 |
| GO:0034641 | cellular nitrogen compound metabolic process | 2.85E-11 | 3.67E-10 |
| GO:0009767 | photosynthetic electron transport chain | 2.97E-11 | 3.77E-10 |
| GO:0009658 | chloroplast organization | 5.87E-11 | 7.35E-10 |
| GO:0051640 | organelle localization | 6.05E-11 | 7.49E-10 |
| GO:0044710 | single-organism metabolic process | 8.74E-11 | 1.07E-09 |
| GO:0018130 | heterocycle biosynthetic process | 1.31E-10 | 1.58E-09 |
| GO:0016070 | RNA metabolic process | 1.66E-10 | 1.98E-09 |
| GO:0016109 | tetraterpenoid biosynthetic process | 2.58E-10 | 3.00E-09 |
| GO:0016117 | carotenoid biosynthetic process | 2.58E-10 | 3.00E-09 |
| GO:1901362 | organic cyclic compound biosynthetic process | 2.74E-10 | 3.14E-09 |
| GO:0016043 | cellular component organization | 3.12E-10 | 3.54E-09 |
| GO:0042793 | transcription from plastid promoter | 3.82E-10 | 4.28E-09 |
| GO:0016108 | tetraterpenoid metabolic process | 4.02E-10 | 4.40E-09 |
| GO:0016116 | carotenoid metabolic process | 4.02E-10 | 4.40E-09 |
| GO:0006725 | cellular aromatic compound metabolic process | 2.45E-09 | 2.66E-08 |
| GO:0071840 | cellular component organization or biogenesis | 3.47E-09 | 3.72E-08 |
| GO:0046483 | heterocycle metabolic process | 3.90E-09 | 4.13E-08 |
| GO:1901360 | organic cyclic compound metabolic process | 5.30E-09 | 5.55E-08 |
| GO:0008152 | metabolic process | 6.62E-09 | 6.85E-08 |
| GO:0006732 | coenzyme metabolic process | 1.03E-08 | 1.05E-07 |
| GO:0043604 | amide biosynthetic process | 1.27E-08 | 1.29E-07 |
| GO:0019362 | pyridine nucleotide metabolic process | 1.89E-08 | 1.88E-07 |
| GO:0046496 | nicotinamide nucleotide metabolic process | 1.89E-08 | 1.88E-07 |
| GO:0072524 | pyridine-containing compound metabolic process | 2.60E-08 | 2.55E-07 |
| GO:0044237 | cellular metabolic process | 3.06E-08 | 2.98E-07 |
| GO:0006396 | RNA processing | 3.61E-08 | 3.47E-07 |
| GO:0034645 | cellular macromolecule biosynthetic process | 5.33E-08 | 5.08E-07 |
| GO:0043603 | cellular amide metabolic process | 8.95E-08 | 8.44E-07 |
| GO:0006412 | translation | 9.85E-08 | 9.21E-07 |
| GO:0043043 | peptide biosynthetic process | 1.12E-07 | 1.04E-06 |
| GO:0006733 | oxidoreduction coenzyme metabolic process | 1.19E-07 | 1.09E-06 |
| GO:0022900 | electron transport chain | 1.23E-07 | 1.12E-06 |
| GO:0005982 | starch metabolic process | 1.77E-07 | 1.59E-06 |
| GO:0000023 | maltose metabolic process | 1.94E-07 | 1.73E-06 |
| GO:0009059 | macromolecule biosynthetic process | 2.03E-07 | 1.79E-06 |
| GO:0090304 | nucleic acid metabolic process | 2.40E-07 | 2.10E-06 |
| GO:0016114 | terpenoid biosynthetic process | 2.59E-07 | 2.24E-06 |
| GO:0006518 | peptide metabolic process | 3.59E-07 | 3.08E-06 |
| GO:0045036 | protein targeting to chloroplast | 3.97E-07 | 3.31E-06 |
| GO:0072596 | establishment of protein localization to chloroplast | 3.97E-07 | 3.31E-06 |
| GO:0072598 | protein localization to chloroplast | 3.97E-07 | 3.31E-06 |
| GO:0006996 | organelle organization | 4.21E-07 | 3.49E-06 |
| GO:0008652 | cellular amino acid biosynthetic process | 4.46E-07 | 3.66E-06 |
| GO:0019252 | starch biosynthetic process | 4.87E-07 | 3.97E-06 |
| GO:0006721 | terpenoid metabolic process | 4.95E-07 | 4.00E-06 |
| GO:0010155 | regulation of proton transport | 7.28E-07 | 5.83E-06 |
| GO:0043933 | macromolecular complex subunit organization | 9.43E-07 | 7.49E-06 |
| GO:0006139 | nucleobase-containing compound metabolic process | 9.90E-07 | 7.79E-06 |
| GO:0016053 | organic acid biosynthetic process | 1.20E-06 | 9.33E-06 |
| GO:0046394 | carboxylic acid biosynthetic process | 1.20E-06 | 9.33E-06 |
| GO:0009070 | serine family amino acid biosynthetic process | 1.57E-06 | 1.21E-05 |
| GO:0010109 | regulation of photosynthesis | 2.03E-06 | 1.55E-05 |
| GO:0009069 | serine family amino acid metabolic process | 2.90E-06 | 2.19E-05 |
| GO:0019344 | cysteine biosynthetic process | 3.65E-06 | 2.74E-05 |
| GO:0010103 | stomatal complex morphogenesis | 4.78E-06 | 3.56E-05 |
| GO:0010218 | response to far red light | 5.49E-06 | 4.06E-05 |
| GO:0006534 | cysteine metabolic process | 5.95E-06 | 4.29E-05 |
| GO:0035303 | regulation of dephosphorylation | 5.95E-06 | 4.29E-05 |
| GO:0035304 | regulation of protein dephosphorylation | 5.95E-06 | 4.29E-05 |
| GO:0010114 | response to red light | 6.45E-06 | 4.62E-05 |
| GO:0006351 | transcription, DNA-templated | 8.38E-06 | 5.96E-05 |
| GO:0044283 | small molecule biosynthetic process | 9.12E-06 | 6.44E-05 |
| GO:0097659 | nucleic acid-templated transcription | 1.12E-05 | 7.88E-05 |
| GO:0009765 | photosynthesis, light harvesting | 1.18E-05 | 8.19E-05 |
| GO:0009637 | response to blue light | 1.20E-05 | 8.28E-05 |
| GO:0032774 | RNA biosynthetic process | 1.32E-05 | 9.04E-05 |
| GO:0005984 | disaccharide metabolic process | 1.34E-05 | 9.12E-05 |
| GO:0006520 | cellular amino acid metabolic process | 1.69E-05 | 1.14E-04 |
| GO:0016226 | iron-sulfur cluster assembly | 3.04E-05 | 2.02E-04 |
| GO:0031163 | metallo-sulfur cluster assembly | 3.04E-05 | 2.02E-04 |
| GO:0010374 | stomatal complex development | 3.18E-05 | 2.11E-04 |
| GO:0010016 | shoot system morphogenesis | 3.48E-05 | 2.29E-04 |
| GO:0016556 | mRNA modification | 4.14E-05 | 2.70E-04 |
| GO:0044272 | sulfur compound biosynthetic process | 4.25E-05 | 2.76E-04 |
| GO:0030091 | protein repair | 4.69E-05 | 3.00E-04 |
| GO:0032543 | mitochondrial translation | 4.69E-05 | 3.00E-04 |
| GO:0043269 | regulation of ion transport | 4.90E-05 | 3.11E-04 |
| GO:0009311 | oligosaccharide metabolic process | 4.97E-05 | 3.14E-04 |
| GO:0019220 | regulation of phosphate metabolic process | 8.46E-05 | 5.27E-04 |
| GO:0051174 | regulation of phosphorus metabolic process | 8.46E-05 | 5.27E-04 |
| GO:0042372 | phylloquinone biosynthetic process | 9.84E-05 | 6.05E-04 |
| GO:0042374 | phylloquinone metabolic process | 9.84E-05 | 6.05E-04 |
| GO:0055086 | nucleobase-containing small molecule metabolic process | 1.14E-04 | 6.98E-04 |
| GO:0006612 | protein targeting to membrane | 1.40E-04 | 8.53E-04 |
| GO:0070125 | mitochondrial translational elongation | 1.46E-04 | 8.83E-04 |
| GO:1901607 | alpha-amino acid biosynthetic process | 1.50E-04 | 8.87E-04 |
| GO:0018160 | peptidyl-pyrromethane cofactor linkage | 1.51E-04 | 8.87E-04 |
| GO:0051775 | response to redox state | 1.51E-04 | 8.87E-04 |
| GO:0071461 | cellular response to redox state | 1.51E-04 | 8.87E-04 |
| GO:0051049 | regulation of transport | 1.61E-04 | 9.40E-04 |
| GO:0019693 | ribose phosphate metabolic process | 1.88E-04 | 1.07E-03 |
| GO:0016143 | S-glycoside metabolic process | 1.88E-04 | 1.07E-03 |
| GO:0019757 | glycosinolate metabolic process | 1.88E-04 | 1.07E-03 |
| GO:0019760 | glucosinolate metabolic process | 1.88E-04 | 1.07E-03 |
| GO:0009696 | salicylic acid metabolic process | 1.98E-04 | 1.12E-03 |
| GO:0009697 | salicylic acid biosynthetic process | 1.98E-04 | 1.12E-03 |
| GO:0000097 | sulfur amino acid biosynthetic process | 2.00E-04 | 1.12E-03 |
| GO:0006073 | cellular glucan metabolic process | 2.25E-04 | 1.25E-03 |
| GO:0009893 | positive regulation of metabolic process | 2.38E-04 | 1.32E-03 |
| GO:0009117 | nucleotide metabolic process | 2.40E-04 | 1.32E-03 |
| GO:0006753 | nucleoside phosphate metabolic process | 2.49E-04 | 1.37E-03 |
| GO:0044042 | glucan metabolic process | 2.51E-04 | 1.37E-03 |
| GO:0006790 | sulfur compound metabolic process | 2.55E-04 | 1.38E-03 |
| GO:0044699 | single-organism process | 2.58E-04 | 1.39E-03 |
| GO:0010206 | photosystem II repair | 2.82E-04 | 1.51E-03 |
| GO:0042537 | benzene-containing compound metabolic process | 3.22E-04 | 1.72E-03 |
| GO:0044763 | single-organism cellular process | 3.27E-04 | 1.73E-03 |
| GO:0046189 | phenol-containing compound biosynthetic process | 3.31E-04 | 1.74E-03 |
| GO:0071704 | organic substance metabolic process | 3.99E-04 | 2.08E-03 |
| GO:0018958 | phenol-containing compound metabolic process | 4.00E-04 | 2.08E-03 |
| GO:0048367 | shoot system development | 4.01E-04 | 2.08E-03 |
| GO:0009595 | detection of biotic stimulus | 4.17E-04 | 2.15E-03 |
| GO:0009987 | cellular process | 4.21E-04 | 2.16E-03 |
| GO:0042548 | regulation of photosynthesis, light reaction | 4.80E-04 | 2.44E-03 |
| GO:0043467 | regulation of generation of precursor metabolites and energy | 4.80E-04 | 2.44E-03 |
| GO:0072657 | protein localization to membrane | 5.26E-04 | 2.64E-03 |
| GO:0090150 | establishment of protein localization to membrane | 5.26E-04 | 2.64E-03 |
| GO:0055114 | oxidation-reduction process | 5.30E-04 | 2.65E-03 |
| GO:0051649 | establishment of localization in cell | 5.45E-04 | 2.70E-03 |
| GO:0090626 | plant epidermis morphogenesis | 5.83E-04 | 2.88E-03 |
| GO:0000096 | sulfur amino acid metabolic process | 6.61E-04 | 3.25E-03 |
| GO:0098542 | defense response to other organism | 7.08E-04 | 3.46E-03 |
| GO:0009814 | defense response, incompatible interaction | 7.16E-04 | 3.48E-03 |
| GO:0009250 | glucan biosynthetic process | 7.92E-04 | 3.83E-03 |
| GO:0006796 | phosphate-containing compound metabolic process | 8.15E-04 | 3.92E-03 |
| GO:0032879 | regulation of localization | 8.27E-04 | 3.96E-03 |
| GO:0016144 | S-glycoside biosynthetic process | 8.51E-04 | 4.01E-03 |
| GO:0019758 | glycosinolate biosynthetic process | 8.51E-04 | 4.01E-03 |
| GO:0019761 | glucosinolate biosynthetic process | 8.51E-04 | 4.01E-03 |
| GO:0010310 | regulation of hydrogen peroxide metabolic process | 8.67E-04 | 4.07E-03 |
| GO:0010258 | NADH dehydrogenase complex (plastoquinone) assembly | 8.90E-04 | 4.14E-03 |
| GO:0080005 | photosystem stoichiometry adjustment | 8.90E-04 | 4.14E-03 |
| GO:0044238 | primary metabolic process | 9.28E-04 | 4.29E-03 |
| GO:0009628 | response to abiotic stimulus | 9.93E-04 | 4.57E-03 |
| GO:0006793 | phosphorus metabolic process | 1.06E-03 | 4.86E-03 |
| GO:0010628 | positive regulation of gene expression | 1.24E-03 | 5.64E-03 |
| GO:1901605 | alpha-amino acid metabolic process | 1.25E-03 | 5.67E-03 |
| GO:0010557 | positive regulation of macromolecule biosynthetic process | 1.27E-03 | 5.74E-03 |
| GO:0044260 | cellular macromolecule metabolic process | 1.47E-03 | 6.60E-03 |
| GO:0031328 | positive regulation of cellular biosynthetic process | 1.49E-03 | 6.65E-03 |
| GO:0051173 | positive regulation of nitrogen compound metabolic process | 1.57E-03 | 6.97E-03 |
| GO:0051641 | cellular localization | 1.59E-03 | 7.04E-03 |
| GO:0006414 | translational elongation | 1.60E-03 | 7.04E-03 |
| GO:0044264 | cellular polysaccharide metabolic process | 1.60E-03 | 7.04E-03 |
| GO:0006636 | unsaturated fatty acid biosynthetic process | 1.73E-03 | 7.47E-03 |
| GO:0009862 | systemic acquired resistance, salicylic acid mediated signaling pathway | 1.73E-03 | 7.47E-03 |
| GO:0033559 | unsaturated fatty acid metabolic process | 1.73E-03 | 7.47E-03 |
| GO:2000377 | regulation of reactive oxygen species metabolic process | 1.73E-03 | 7.47E-03 |
| GO:0034654 | nucleobase-containing compound biosynthetic process | 1.90E-03 | 8.16E-03 |
| GO:0009891 | positive regulation of biosynthetic process | 2.06E-03 | 8.81E-03 |
| GO:0045893 | positive regulation of transcription, DNA-templated | 2.11E-03 | 8.89E-03 |
| GO:1902680 | positive regulation of RNA biosynthetic process | 2.11E-03 | 8.89E-03 |
| GO:1903508 | positive regulation of nucleic acid-templated transcription | 2.11E-03 | 8.89E-03 |
| GO:0043207 | response to external biotic stimulus | 2.17E-03 | 9.04E-03 |
| GO:0051707 | response to other organism | 2.17E-03 | 9.04E-03 |
| GO:0010257 | NADH dehydrogenase complex assembly | 2.19E-03 | 9.04E-03 |
| GO:0018065 | protein-cofactor linkage | 2.19E-03 | 9.04E-03 |
| GO:0045038 | protein import into chloroplast thylakoid membrane | 2.19E-03 | 9.04E-03 |
| GO:0051254 | positive regulation of RNA metabolic process | 2.34E-03 | 9.63E-03 |
| GO:0048518 | positive regulation of biological process | 2.40E-03 | 9.84E-03 |
| GO:0043900 | regulation of multi-organism process | 2.85E-03 | 1.16E-02 |
| GO:0045935 | positive regulation of nucleobase-containing compound metabolic process | 2.88E-03 | 1.17E-02 |
| GO:0031399 | regulation of protein modification process | 2.98E-03 | 1.20E-02 |
| GO:0002376 | immune system process | 3.00E-03 | 1.21E-02 |
| GO:0031425 | chloroplast RNA processing | 3.04E-03 | 1.22E-02 |
| GO:0009886 | post-embryonic morphogenesis | 3.34E-03 | 1.33E-02 |
| GO:0009416 | response to light stimulus | 3.37E-03 | 1.34E-02 |
| GO:0009790 | embryo development | 3.95E-03 | 1.56E-02 |
| GO:0009793 | embryo development ending in seed dormancy | 3.97E-03 | 1.56E-02 |
| GO:0009607 | response to biotic stimulus | 4.33E-03 | 1.70E-02 |
| GO:0044262 | cellular carbohydrate metabolic process | 4.41E-03 | 1.72E-02 |
| GO:0009791 | post-embryonic development | 4.69E-03 | 1.83E-02 |
| GO:0044550 | secondary metabolite biosynthetic process | 4.83E-03 | 1.87E-02 |
| GO:0010200 | response to chitin | 4.98E-03 | 1.91E-02 |
| GO:0010243 | response to organonitrogen compound | 4.98E-03 | 1.91E-02 |
| GO:0006782 | protoporphyrinogen IX biosynthetic process | 5.13E-03 | 1.96E-02 |
| GO:0046501 | protoporphyrinogen IX metabolic process | 5.13E-03 | 1.96E-02 |
| GO:0006783 | heme biosynthetic process | 5.31E-03 | 2.02E-02 |
| GO:0033692 | cellular polysaccharide biosynthetic process | 5.36E-03 | 2.03E-02 |
| GO:0048731 | system development | 5.89E-03 | 2.22E-02 |
| GO:0009867 | jasmonic acid mediated signaling pathway | 5.99E-03 | 2.24E-02 |
| GO:0071395 | cellular response to jasmonic acid stimulus | 5.99E-03 | 2.24E-02 |
| GO:0045087 | innate immune response | 6.10E-03 | 2.27E-02 |
| GO:0006955 | immune response | 6.33E-03 | 2.32E-02 |
| GO:0000165 | MAPK cascade | 6.36E-03 | 2.32E-02 |
| GO:0009772 | photosynthetic electron transport in photosystem II | 6.36E-03 | 2.32E-02 |
| GO:0018198 | peptidyl-cysteine modification | 6.36E-03 | 2.32E-02 |
| GO:0032544 | plastid translation | 6.36E-03 | 2.32E-02 |
| GO:0009073 | aromatic amino acid family biosynthetic process | 6.39E-03 | 2.32E-02 |
| GO:0072330 | monocarboxylic acid biosynthetic process | 6.45E-03 | 2.34E-02 |
| GO:0050832 | defense response to fungus | 6.67E-03 | 2.39E-02 |
| GO:0043085 | positive regulation of catalytic activity | 6.68E-03 | 2.39E-02 |
| GO:0044093 | positive regulation of molecular function | 6.68E-03 | 2.39E-02 |
| GO:0006352 | DNA-templated transcription, initiation | 6.74E-03 | 2.41E-02 |
| GO:0009108 | coenzyme biosynthetic process | 6.79E-03 | 2.41E-02 |
| GO:0042181 | ketone biosynthetic process | 7.02E-03 | 2.46E-02 |
| GO:1901661 | quinone metabolic process | 7.02E-03 | 2.46E-02 |
| GO:1901663 | quinone biosynthetic process | 7.02E-03 | 2.46E-02 |
| GO:0009314 | response to radiation | 7.12E-03 | 2.46E-02 |
| GO:0006354 | DNA-templated transcription, elongation | 7.14E-03 | 2.46E-02 |
| GO:0009863 | salicylic acid mediated signaling pathway | 7.14E-03 | 2.46E-02 |
| GO:0010363 | regulation of plant-type hypersensitive response | 7.14E-03 | 2.46E-02 |
| GO:0031348 | negative regulation of defense response | 7.14E-03 | 2.46E-02 |
| GO:0071446 | cellular response to salicylic acid stimulus | 7.14E-03 | 2.46E-02 |
| GO:0019748 | secondary metabolic process | 7.31E-03 | 2.51E-02 |
| GO:0051606 | detection of stimulus | 7.55E-03 | 2.58E-02 |
| GO:0090558 | plant epidermis development | 7.63E-03 | 2.60E-02 |
| GO:0048316 | seed development | 7.86E-03 | 2.66E-02 |
| GO:0042168 | heme metabolic process | 8.31E-03 | 2.81E-02 |
| GO:0010154 | fruit development | 8.55E-03 | 2.88E-02 |
| GO:0046777 | protein autophosphorylation | 8.57E-03 | 2.88E-02 |
| GO:0009751 | response to salicylic acid | 8.88E-03 | 2.96E-02 |
| GO:0009743 | response to carbohydrate | 8.88E-03 | 2.96E-02 |
| GO:0010604 | positive regulation of macromolecule metabolic process | 9.32E-03 | 3.10E-02 |
| GO:0006605 | protein targeting | 9.66E-03 | 3.18E-02 |
| GO:0032268 | regulation of cellular protein metabolic process | 9.66E-03 | 3.18E-02 |
| GO:0019216 | regulation of lipid metabolic process | 1.05E-02 | 3.45E-02 |
| GO:0009627 | systemic acquired resistance | 1.09E-02 | 3.56E-02 |
| GO:0051246 | regulation of protein metabolic process | 1.11E-02 | 3.62E-02 |
| GO:0009753 | response to jasmonic acid | 1.14E-02 | 3.71E-02 |
| GO:0031325 | positive regulation of cellular metabolic process | 1.15E-02 | 3.73E-02 |
| GO:0006729 | tetrahydrobiopterin biosynthetic process | 1.23E-02 | 3.75E-02 |
| GO:0009118 | regulation of nucleoside metabolic process | 1.23E-02 | 3.75E-02 |
| GO:0010322 | regulation of isopentenyl diphosphate biosynthetic process, methylerythritol 4-phosphate pathway | 1.23E-02 | 3.75E-02 |
| GO:0010323 | negative regulation of isopentenyl diphosphate biosynthetic process, methylerythritol 4-phosphate pathway | 1.23E-02 | 3.75E-02 |
| GO:0010677 | negative regulation of cellular carbohydrate metabolic process | 1.23E-02 | 3.75E-02 |
| GO:0018023 | peptidyl-lysine trimethylation | 1.23E-02 | 3.75E-02 |
| GO:0030808 | regulation of nucleotide biosynthetic process | 1.23E-02 | 3.75E-02 |
| GO:0035970 | peptidyl-threonine dephosphorylation | 1.23E-02 | 3.75E-02 |
| GO:0045912 | negative regulation of carbohydrate metabolic process | 1.23E-02 | 3.75E-02 |
| GO:0046146 | tetrahydrobiopterin metabolic process | 1.23E-02 | 3.75E-02 |
| GO:0051592 | response to calcium ion | 1.23E-02 | 3.75E-02 |
| GO:0071071 | regulation of phospholipid biosynthetic process | 1.23E-02 | 3.75E-02 |
| GO:0071072 | negative regulation of phospholipid biosynthetic process | 1.23E-02 | 3.75E-02 |
| GO:0071277 | cellular response to calcium ion | 1.23E-02 | 3.75E-02 |
| GO:0080020 | regulation of coenzyme A biosynthetic process | 1.23E-02 | 3.75E-02 |
| GO:1900371 | regulation of purine nucleotide biosynthetic process | 1.23E-02 | 3.75E-02 |
| GO:1900542 | regulation of purine nucleotide metabolic process | 1.23E-02 | 3.75E-02 |
| GO:1903725 | regulation of phospholipid metabolic process | 1.23E-02 | 3.75E-02 |
| GO:1903726 | negative regulation of phospholipid metabolic process | 1.23E-02 | 3.75E-02 |
| GO:0006528 | asparagine metabolic process | 1.25E-02 | 3.77E-02 |
| GO:0006529 | asparagine biosynthetic process | 1.25E-02 | 3.77E-02 |
| GO:0006952 | defense response | 1.25E-02 | 3.78E-02 |
| GO:0034637 | cellular carbohydrate biosynthetic process | 1.29E-02 | 3.89E-02 |
| GO:0043170 | macromolecule metabolic process | 1.35E-02 | 4.05E-02 |
| GO:0000271 | polysaccharide biosynthetic process | 1.36E-02 | 4.05E-02 |
| GO:1902580 | single-organism cellular localization | 1.36E-02 | 4.05E-02 |
| GO:0045088 | regulation of innate immune response | 1.38E-02 | 4.07E-02 |
| GO:0050776 | regulation of immune response | 1.38E-02 | 4.07E-02 |
| GO:1901700 | response to oxygen-containing compound | 1.40E-02 | 4.12E-02 |
| GO:0009605 | response to external stimulus | 1.40E-02 | 4.13E-02 |
| GO:0005976 | polysaccharide metabolic process | 1.42E-02 | 4.15E-02 |
| GO:0042335 | cuticle development | 1.43E-02 | 4.17E-02 |
| GO:0080135 | regulation of cellular response to stress | 1.50E-02 | 4.38E-02 |
| GO:0006631 | fatty acid metabolic process | 1.52E-02 | 4.40E-02 |
| GO:0002682 | regulation of immune system process | 1.57E-02 | 4.54E-02 |
| GO:0009072 | aromatic amino acid family metabolic process | 1.61E-02 | 4.64E-02 |
| GO:0000038 | very long-chain fatty acid metabolic process | 1.62E-02 | 4.65E-02 |
| GO:0000413 | protein peptidyl-prolyl isomerization | 1.71E-02 | 4.90E-02 |
| **Down-regulated genes** | | |  |
| GO:2000022 | regulation of jasmonic acid mediated signaling pathway | 1.72E-09 | 1.40E-06 |
| GO:0009611 | response to wounding | 5.27E-06 | 2.16E-03 |
| GO:0031347 | regulation of defense response | 2.06E-05 | 5.62E-03 |
| GO:0080134 | regulation of response to stress | 6.70E-05 | 1.37E-02 |
| GO:0009966 | regulation of signal transduction | 2.47E-04 | 3.42E-02 |
| GO:0023051 | regulation of signaling | 2.76E-04 | 3.42E-02 |
| GO:0010646 | regulation of cell communication | 2.92E-04 | 3.42E-02 |

Table S10 List of GO analysis (**CC**) for the specific common DEGs of 141 and DH40 (GO terms shown were significant at FDR ≤ 0.05 for up-regulated genes, and P-value ≤ 0.05 for down-regulated genes).

| **GO ID** | **GO Term** | **P-value** | **FDR** |
| --- | --- | --- | --- |
| **Up-regulated genes** | |  |  |
| GO:0009507 | chloroplast | 7.68E-50 | 1.20E-47 |
| GO:0009536 | plastid | 3.60E-48 | 2.81E-46 |
| GO:0044434 | chloroplast part | 8.41E-46 | 4.37E-44 |
| GO:0044435 | plastid part | 2.23E-45 | 8.69E-44 |
| GO:0009579 | thylakoid | 9.33E-42 | 2.91E-40 |
| GO:0009532 | plastid stroma | 4.03E-33 | 1.05E-31 |
| GO:0009570 | chloroplast stroma | 2.01E-32 | 4.47E-31 |
| GO:0044436 | thylakoid part | 8.56E-30 | 1.67E-28 |
| GO:0034357 | photosynthetic membrane | 9.10E-28 | 1.58E-26 |
| GO:0009534 | chloroplast thylakoid | 4.11E-27 | 5.83E-26 |
| GO:0031976 | plastid thylakoid | 4.11E-27 | 5.83E-26 |
| GO:0009941 | chloroplast envelope | 4.22E-23 | 5.49E-22 |
| GO:0009526 | plastid envelope | 1.30E-22 | 1.57E-21 |
| GO:0031984 | organelle subcompartment | 2.14E-21 | 2.35E-20 |
| GO:0042651 | thylakoid membrane | 2.26E-21 | 2.35E-20 |
| GO:0044444 | cytoplasmic part | 1.48E-18 | 1.45E-17 |
| GO:0009535 | chloroplast thylakoid membrane | 1.16E-16 | 1.01E-15 |
| GO:0055035 | plastid thylakoid membrane | 1.16E-16 | 1.01E-15 |
| GO:0009521 | photosystem | 2.21E-15 | 1.81E-14 |
| GO:0031967 | organelle envelope | 7.28E-15 | 5.68E-14 |
| GO:0031975 | envelope | 9.38E-15 | 6.97E-14 |
| GO:0005737 | cytoplasm | 2.27E-13 | 1.61E-12 |
| GO:0044446 | intracellular organelle part | 2.73E-10 | 1.85E-09 |
| GO:0044422 | organelle part | 3.52E-10 | 2.29E-09 |
| GO:0009522 | photosystem I | 8.06E-09 | 5.03E-08 |
| GO:0005840 | ribosome | 3.93E-08 | 2.36E-07 |
| GO:0009538 | photosystem I reaction center | 5.47E-08 | 3.16E-07 |
| GO:0031977 | thylakoid lumen | 8.39E-08 | 4.68E-07 |
| GO:0009523 | photosystem II | 2.11E-07 | 1.13E-06 |
| GO:0044424 | intracellular part | 3.30E-07 | 1.71E-06 |
| GO:0098796 | membrane protein complex | 5.13E-07 | 2.58E-06 |
| GO:0030529 | intracellular ribonucleoprotein complex | 8.26E-07 | 3.91E-06 |
| GO:1990904 | ribonucleoprotein complex | 8.26E-07 | 3.91E-06 |
| GO:0009654 | photosystem II oxygen evolving complex | 1.77E-06 | 8.13E-06 |
| GO:0005622 | intracellular | 2.47E-06 | 1.10E-05 |
| GO:0010287 | plastoglobule | 3.35E-06 | 1.45E-05 |
| GO:0005623 | cell | 6.87E-06 | 2.90E-05 |
| GO:0032991 | macromolecular complex | 7.42E-06 | 3.04E-05 |
| GO:0044464 | cell part | 9.87E-06 | 3.95E-05 |
| GO:0043231 | intracellular membrane-bounded organelle | 2.04E-05 | 7.94E-05 |
| GO:0043227 | membrane-bounded organelle | 2.18E-05 | 8.28E-05 |
| GO:0043229 | intracellular organelle | 5.38E-05 | 2.00E-04 |
| GO:0009295 | nucleoid | 5.77E-05 | 2.09E-04 |
| GO:0043226 | organelle | 6.34E-05 | 2.25E-04 |
| GO:0005759 | mitochondrial matrix | 1.61E-04 | 5.57E-04 |
| GO:0000313 | organellar ribosome | 4.36E-04 | 1.48E-03 |
| GO:1990204 | oxidoreductase complex | 4.66E-04 | 1.55E-03 |
| GO:0009543 | chloroplast thylakoid lumen | 5.52E-04 | 1.72E-03 |
| GO:0010007 | magnesium chelatase complex | 5.52E-04 | 1.72E-03 |
| GO:0031978 | plastid thylakoid lumen | 5.52E-04 | 1.72E-03 |
| GO:0010598 | NAD(P)H dehydrogenase complex (plastoquinone) | 6.50E-04 | 1.99E-03 |
| GO:0019013 | viral nucleocapsid | 7.00E-04 | 2.10E-03 |
| GO:0019028 | viral capsid | 1.07E-03 | 3.14E-03 |
| GO:0019012 | virion | 1.25E-03 | 3.54E-03 |
| GO:0044423 | virion part | 1.25E-03 | 3.54E-03 |
| GO:0005761 | mitochondrial ribosome | 1.74E-03 | 4.84E-03 |
| GO:0043228 | non-membrane-bounded organelle | 3.43E-03 | 9.24E-03 |
| GO:0043232 | intracellular non-membrane-bounded organelle | 3.43E-03 | 9.24E-03 |
| GO:0005739 | mitochondrion | 3.80E-03 | 1.00E-02 |
| GO:0048046 | apoplast | 4.96E-03 | 1.29E-02 |
| GO:0005762 | mitochondrial large ribosomal subunit | 6.40E-03 | 1.64E-02 |
| GO:0000314 | organellar small ribosomal subunit | 7.78E-03 | 1.96E-02 |
| GO:0000315 | organellar large ribosomal subunit | 8.53E-03 | 2.11E-02 |
| GO:0009782 | photosystem I antenna complex | 1.37E-02 | 3.33E-02 |
| GO:0019898 | extrinsic component of membrane | 1.69E-02 | 4.06E-02 |
| **Down-regulated genes** | | |  |
| GO:0016021 | integral component of membrane | 7.60E-04 | 7.19E-02 |
| GO:0031224 | intrinsic component of membrane | 9.99E-04 | 7.19E-02 |
| GO:0044425 | membrane part | 2.51E-03 | 1.20E-01 |
| GO:0016020 | membrane | 5.90E-03 | 2.12E-01 |
| GO:0032039 | integrator complex | 1.95E-02 | 5.62E-01 |
| GO:0017053 | transcriptional repressor complex | 2.91E-02 | 6.15E-01 |
| GO:0005886 | plasma membrane | 2.99E-02 | 6.15E-01 |
| GO:0046658 | anchored component of plasma membrane | 4.52E-02 | 8.13E-01 |

Table S11 List of GO analysis (**MF**) for the specific common DEGs of 141 and DH40 (All GO terms shown were significant at FDR ≤ 0.05).

| **GO ID** | **GO Term** | **P-value** | **FDR** |
| --- | --- | --- | --- |
| **Up-regulated genes** | |  |  |
| GO:0003735 | structural constituent of ribosome | 6.76E-07 | 1.05E-04 |
| GO:0000996 | core DNA-dependent RNA polymerase binding promoter specificity activity | 9.27E-07 | 1.05E-04 |
| GO:0016987 | sigma factor activity | 9.27E-07 | 1.05E-04 |
| GO:0000990 | transcription factor activity, core RNA polymerase binding | 2.73E-06 | 2.32E-04 |
| GO:0001053 | plastid sigma factor activity | 5.07E-06 | 3.43E-04 |
| GO:0005198 | structural molecule activity | 1.13E-05 | 6.41E-04 |
| GO:0004418 | hydroxymethylbilane synthase activity | 1.18E-04 | 5.73E-03 |
| GO:0003746 | translation elongation factor activity | 3.87E-04 | 1.64E-02 |
| GO:0016851 | magnesium chelatase activity | 7.00E-04 | 2.08E-02 |
| GO:0051002 | ligase activity, forming nitrogen-metal bonds | 7.00E-04 | 2.08E-02 |
| GO:0051003 | ligase activity, forming nitrogen-metal bonds, forming coordination complexes | 7.00E-04 | 2.08E-02 |
| GO:0003723 | RNA binding | 7.36E-04 | 2.08E-02 |
| GO:0016168 | chlorophyll binding | 8.37E-04 | 2.18E-02 |
| **Down-regulated genes** | | |  |
| GO:0003714 | transcription corepressor activity | 5.65E-08 | 1.82E-05 |
| GO:0003712 | transcription cofactor activity | 2.74E-05 | 4.42E-03 |
| GO:0000989 | transcription factor activity, transcription factor binding | 6.66E-05 | 7.15E-03 |
| GO:0000988 | transcription factor activity, protein binding | 1.22E-04 | 9.79E-03 |
