# Supplementary material for: Transcriptome sequencing analysis of maize embryonic callus during early redifferentiation
Source: BMC Genomics. 2019 Feb 27;20:159. doi: 10.1186/s12864-019-5506-7 (PMC6391841; doi:10.1186/s12864-019-5506-7)
Supplement: Supplementary file 8 — Table S12. List of GO analysis (BP) for the specific common DEGs of DH3732 and ZYDH381–1 (All GO terms shown were significant at FDR ≤ 0.05); Table S13. List of GO analysis (CC) for the specific common DEGs of DH3732 and ZYDH381–1 (All GO terms shown were significant at FDR ≤ 0.05); Table S14. List of GO analysis (MF) for the specific common DEGs of DH3732 and ZYDH381–1 (All GO terms shown were significant at FDR ≤ 0.05).). (DOCX 20 kb) [file 12864_2019_5506_MOESM8_ESM.docx]

Table S12 List of GO analysis (**BP**) for the specific common DEGs of DH3732 and ZYDH381-1 (All GO terms shown were significant at FDR ≤ 0.05).

| **GO ID** | **GO Term** | **P-value** | **FDR** |
| --- | --- | --- | --- |
| **Up-regulated genes** | |  |  |
| GO:0006334 | nucleosome assembly | 2.16E-08 | 2.21E-05 |
| GO:0034728 | nucleosome organization | 3.75E-08 | 2.21E-05 |
| GO:0065004 | protein-DNA complex assembly | 1.02E-07 | 4.00E-05 |
| GO:0071824 | protein-DNA complex subunit organization | 1.53E-07 | 4.50E-05 |
| GO:0031497 | chromatin assembly | 3.91E-07 | 9.21E-05 |
| GO:0071103 | DNA conformation change | 5.88E-07 | 1.16E-04 |
| GO:0006323 | DNA packaging | 9.69E-07 | 1.63E-04 |
| GO:0006333 | chromatin assembly or disassembly | 2.20E-06 | 3.24E-04 |
| GO:0055114 | oxidation-reduction process | 1.01E-05 | 1.32E-03 |
| GO:0072330 | monocarboxylic acid biosynthetic process | 3.49E-05 | 4.11E-03 |
| GO:0006633 | fatty acid biosynthetic process | 1.43E-04 | 1.53E-02 |
| **Down-regulated genes** | |  |  |
| GO:0019219 | regulation of nucleobase-containing compound metabolic process | 1.37E-10 | 1.00E-07 |
| GO:0051252 | regulation of RNA metabolic process | 2.60E-10 | 1.00E-07 |
| GO:1903506 | regulation of nucleic acid-templated transcription | 4.05E-10 | 1.00E-07 |
| GO:2001141 | regulation of RNA biosynthetic process | 4.05E-10 | 1.00E-07 |
| GO:0006355 | regulation of transcription, DNA-templated | 6.15E-10 | 1.22E-07 |
| GO:0050794 | regulation of cellular process | 1.28E-09 | 2.12E-07 |
| GO:0051171 | regulation of nitrogen compound metabolic process | 2.18E-09 | 3.09E-07 |
| GO:0009889 | regulation of biosynthetic process | 3.88E-09 | 4.82E-07 |
| GO:0010556 | regulation of macromolecule biosynthetic process | 5.96E-09 | 6.57E-07 |
| GO:0010468 | regulation of gene expression | 7.55E-09 | 6.81E-07 |
| GO:0031326 | regulation of cellular biosynthetic process | 7.55E-09 | 6.81E-07 |
| GO:2000112 | regulation of cellular macromolecule biosynthetic process | 8.95E-09 | 7.40E-07 |
| GO:0065007 | biological regulation | 1.59E-08 | 1.21E-06 |
| GO:0080090 | regulation of primary metabolic process | 1.29E-07 | 8.59E-06 |
| GO:0050789 | regulation of biological process | 1.30E-07 | 8.59E-06 |
| GO:0031323 | regulation of cellular metabolic process | 1.59E-07 | 9.88E-06 |
| GO:0060255 | regulation of macromolecule metabolic process | 3.29E-07 | 1.92E-05 |
| GO:0009734 | auxin-activated signaling pathway | 1.24E-06 | 6.47E-05 |
| GO:0071365 | cellular response to auxin stimulus | 1.24E-06 | 6.47E-05 |
| GO:0019222 | regulation of metabolic process | 7.69E-06 | 3.82E-04 |
| GO:0009733 | response to auxin | 1.37E-05 | 6.48E-04 |
| GO:0023052 | signaling | 2.90E-05 | 1.31E-03 |
| GO:0007165 | signal transduction | 4.96E-05 | 2.14E-03 |
| GO:0044700 | single organism signaling | 7.04E-05 | 2.91E-03 |
| GO:0000024 | maltose biosynthetic process | 1.99E-04 | 7.72E-03 |
| GO:0009725 | response to hormone | 2.02E-04 | 7.72E-03 |
| GO:0007154 | cell communication | 2.33E-04 | 8.57E-03 |
| GO:0046351 | disaccharide biosynthetic process | 3.73E-04 | 1.32E-02 |
| GO:0009719 | response to endogenous stimulus | 3.87E-04 | 1.33E-02 |
| GO:0009755 | hormone-mediated signaling pathway | 5.46E-04 | 1.70E-02 |
| GO:0032870 | cellular response to hormone stimulus | 5.46E-04 | 1.70E-02 |
| GO:0071495 | cellular response to endogenous stimulus | 5.46E-04 | 1.70E-02 |
| GO:0009312 | oligosaccharide biosynthetic process | 7.45E-04 | 2.24E-02 |
| GO:0016106 | sesquiterpenoid biosynthetic process | 9.04E-04 | 2.64E-02 |
| GO:0051716 | cellular response to stimulus | 9.94E-04 | 2.82E-02 |
| GO:0010033 | response to organic substance | 1.03E-03 | 2.84E-02 |
| GO:1901334 | lactone metabolic process | 1.17E-03 | 2.90E-02 |
| GO:1901336 | lactone biosynthetic process | 1.17E-03 | 2.90E-02 |
| GO:1901600 | strigolactone metabolic process | 1.17E-03 | 2.90E-02 |
| GO:1901601 | strigolactone biosynthetic process | 1.17E-03 | 2.90E-02 |
| GO:0006714 | sesquiterpenoid metabolic process | 1.44E-03 | 3.49E-02 |
| GO:0050896 | response to stimulus | 1.91E-03 | 4.15E-02 |
| GO:0006145 | purine nucleobase catabolic process | 1.93E-03 | 4.15E-02 |
| GO:0006542 | glutamine biosynthetic process | 1.93E-03 | 4.15E-02 |
| GO:0009115 | xanthine catabolic process | 1.93E-03 | 4.15E-02 |
| GO:0046110 | xanthine metabolic process | 1.93E-03 | 4.15E-02 |
| GO:0071482 | cellular response to light stimulus | 1.96E-03 | 4.15E-02 |

Table S13 List of GO analysis (**CC**) for the specific common DEGs of DH3732 and ZYDH381-1 (All GO terms shown were significant at FDR ≤ 0.05).

| **GO ID** | **GO Term** | **P-value** | **FDR** |
| --- | --- | --- | --- |
| **Up-regulated genes** | |  |  |
| GO:0032993 | protein-DNA complex | 2.42E-24 | 2.66E-22 |
| GO:0000786 | nucleosome | 2.94E-24 | 2.66E-22 |
| GO:0044815 | DNA packaging complex | 7.21E-24 | 4.35E-22 |
| GO:0000785 | chromatin | 1.54E-19 | 6.95E-18 |
| GO:0044427 | chromosomal part | 4.08E-18 | 1.48E-16 |
| GO:0005694 | chromosome | 4.64E-16 | 1.40E-14 |
| GO:0000788 | nuclear nucleosome | 8.95E-06 | 2.31E-04 |
| GO:0000790 | nuclear chromatin | 1.40E-04 | 3.18E-03 |
| GO:0044454 | nuclear chromosome part | 1.83E-04 | 3.68E-03 |
| GO:0000228 | nuclear chromosome | 4.07E-04 | 6.19E-03 |
| GO:0043228 | non-membrane-bounded organelle | 4.10E-04 | 6.19E-03 |
| GO:0043232 | intracellular non-membrane-bounded organelle | 4.10E-04 | 6.19E-03 |
| GO:0005618 | cell wall | 1.17E-03 | 1.51E-02 |
| GO:0030312 | external encapsulating structure | 1.17E-03 | 1.51E-02 |
| GO:0005576 | extracellular region | 1.32E-03 | 1.59E-02 |
| GO:0048046 | apoplast | 2.55E-03 | 2.88E-02 |
| **Down-regulated genes** | |  |  |
| GO:0005634 | nucleus | 6.40E-06 | 8.33E-04 |

Table S14 List of GO analysis (**MF**) for the specific common DEGs of DH3732 and ZYDH381-1 (All GO terms shown were significant at FDR ≤ 0.05).

| **GO ID** | **GO Term** | **P-value** | **FDR** |
| --- | --- | --- | --- |
| **Up-regulated genes** | |  |  |
| GO:0046982 | protein heterodimerization activity | 5.27E-21 | 2.09E-18 |
| GO:0046983 | protein dimerization activity | 1.75E-08 | 3.47E-06 |
| GO:0046906 | tetrapyrrole binding | 3.66E-05 | 4.84E-03 |
| GO:0020037 | heme binding | 6.68E-05 | 6.63E-03 |
| GO:0016661 | oxidoreductase activity, acting on other nitrogenous compounds as donors | 9.41E-05 | 7.46E-03 |
| GO:0016491 | oxidoreductase activity | 1.13E-04 | 7.46E-03 |
| GO:0008422 | beta-glucosidase activity | 4.15E-04 | 2.12E-02 |
| GO:0015925 | galactosidase activity | 4.27E-04 | 2.12E-02 |
| GO:0004553 | hydrolase activity, hydrolyzing O-glycosyl compounds | 6.75E-04 | 2.98E-02 |
| GO:0004565 | beta-galactosidase activity | 1.17E-03 | 4.24E-02 |
| GO:0016662 | oxidoreductase activity, acting on other nitrogenous compounds as donors, cytochrome as acceptor | 1.39E-03 | 4.24E-02 |
| GO:0046577 | long-chain-alcohol oxidase activity | 1.39E-03 | 4.24E-02 |
| GO:0050421 | nitrite reductase (NO-forming) activity | 1.39E-03 | 4.24E-02 |
| GO:0016798 | hydrolase activity, acting on glycosyl bonds | 1.51E-03 | 4.28E-02 |
| **Down-regulated genes** | |  |  |
| GO:0003677 | DNA binding | 1.06E-06 | 3.73E-04 |
| GO:0001071 | nucleic acid binding transcription factor activity | 9.76E-06 | 1.15E-03 |
| GO:0003700 | transcription factor activity, sequence-specific DNA binding | 9.76E-06 | 1.15E-03 |
| GO:0001871 | pattern binding | 2.20E-04 | 1.55E-02 |
| GO:0030247 | polysaccharide binding | 2.20E-04 | 1.55E-02 |
| GO:0009881 | photoreceptor activity | 4.47E-04 | 2.63E-02 |
