# Supplementary material for: Transcriptome sequencing analysis of maize embryonic callus during early redifferentiation
Source: BMC Genomics. 2019 Feb 27;20:159. doi: 10.1186/s12864-019-5506-7 (PMC6391841; doi:10.1186/s12864-019-5506-7)
Supplement: Supplementary file 11 — Table S17. List of DEGs related to embryonic callus regeneration. (DOCX 20 kb) [file 12864_2019_5506_MOESM11_ESM.docx]

Table S17 List of DEGs related to embryonic callus regeneration.

| **Gene ID** | **Gene function** |
| --- | --- |
| **Photosynthesis** |  |
| Zm00001d020877 | Photosystem I subunit V (psaG) |
| Zm00001d034543 | Photosystem I subunit II (psaD) |
| Zm00001d019518 | Photosystem I subunit IV (psaE) |
| Zm00001d013146 | Photosystem I subunit III (psaF) |
| Zm00001d034283 | Photosystem I subunit III (psaF) |
| Zm00001d039687 | Photosystem I subunit XI (psaL) |
| Zm00001d014564 | Photosystem II oxygen-evolving enhancer protein 1 (psbO) |
| Zm00001d011362 | Photosystem II oxygen-evolving enhancer protein 2 (psbP) |
| Zm00001d030638 | Photosystem II oxygen-evolving enhancer protein 2 (psbP) |
| Zm00001d049387 | Photosystem II 10kDa protein (psbR) |
| Zm00001d042178 | Photosystem II 13kDa protein (psb28) |
| Zm00001d011833 | Ferredoxin--NADP+ reductase (petH) |
| Zm00001d047789 | F-type H+-transporting ATPase subunit b,ATPF0B (atpF) |
| Zm00001d018069 | Photosynthethic electron transport,ATPF1D (atpH) |
| **Porphyrin and chlorophyll metabolism** | |
| Zm00001d029074 | Uroporphyrinogen decarboxylase,hemE (UROD) |
| Zm00001d015366 | Hydroxymethylbilane synthase,hemC (HMBS) |
| Zm00001d053807 | Hydroxymethylbilane synthase,hemC (HMBS) |
| Zm00001d038547 | Glutamate-1-semialdehyde 2,1-aminomutase (hemL) |
| Zm00001d008681 | Cytochrome c oxidase assembly protein subunit 15 (COX15) |
| Zm00001d022464 | Cytochrome c oxidase assembly protein subunit 15 (COX15) |
| Zm00001d026405 | Glutamyl-tRNA reductase (hemA) |
| Zm00001d026603 | Magnesium chelatase subunit H,chlH (bchH) |
| Zm00001d013013 | Magnesium chelatase subunit H,chlH (bchH) |
| Zm00001d008230 | Magnesium-protoporphyrin IX monomethyl ester (oxidative) cyclase,acsF (chlE) |
| Zm00001d034523 | Pheophorbide a oxygenase,PAO (ACD1) |
| Zm00001d031997 | Divinyl chlorophyllide a 8-vinyl-reductase (DVR) |
| **Photosynthesis - antenna proteins** | |
| Zm00001d006587 | Light-harvesting complex II chlorophyll a/b binding protein 4 (LHCB4) |
| Zm00001d018157 | Light-harvesting complex I chlorophyll a/b binding protein 5 (LHCA5) |
| Zm00001d021906 | Light-harvesting complex I chlorophyll a/b binding protein 2 (LHCA2) |
| **Circadian rhythm – plant** | |
| Zm00001d012703 | Dof zinc finger protein DOF5.5 (CDF1) |
| Zm00001d014198 | Dof zinc finger protein DOF5.5 (CDF1) |
| Zm00001d022525 | Dof zinc finger protein DOF5.5 (CDF1) |
| Zm00001d027846 | Dof zinc finger protein DOF5.5 (CDF1) |
| Zm00001d032999 | Dof zinc finger protein DOF5.5 (CDF1) |
| Zm00001d040362 | Dof zinc finger protein DOF5.5 (CDF1) |
| Zm00001d051700 | Dof zinc finger protein DOF5.5 (CDF1) |
| Zm00001d017176 | Zinc finger protein CONSTANS (CO) |
| Zm00001d017642 | MYB-related transcription factor LHY (LHY) |
| Zm00001d006212 | Pseudo-response regulator 5 (PRR5) |
| Zm00001d017885 | Pseudo-response regulator 1,TOC1 (APRR1) |
| Zm00001d040536 | Phytochrome-interacting factor 3 (PIF3) |
| Zm00001d044232 | Protein EARLY FLOWERING 3 (ELF3) |
| Zm00001d047632 | Phytochrome B (PHYB) |
| Zm00001d033799 | Phytochrome A (PHYA) |
| Zm00001d003477 | Cryptochrome 1 (CRY1) |
| Zm00001d016915 | Cryptochrome 1 (CRY1) |
| **Plant hormone signal transduction** | |
| Zm00001d013370 | Transcription factor MYC2 (MYC2) |
| Zm00001d024522 | Transcription factor MYC2 (MYC2) |
| Zm00001d025141 | Transcription factor MYC2 (MYC2) |
| Zm00001d043706 | Transcription factor MYC2 (MYC2) |
| Zm00001d005813 | Jasmonate ZIM domain-containing protein (JAZ) |
| Zm00001d019692 | Jasmonate ZIM domain-containing protein (JAZ) |
| Zm00001d020614 | Jasmonate ZIM domain-containing protein (JAZ) |
| Zm00001d027899 | Jasmonate ZIM domain-containing protein (JAZ) |
| Zm00001d027900 | Jasmonate ZIM domain-containing protein (JAZ) |
| Zm00001d033049 | Jasmonate ZIM domain-containing protein (JAZ) |
| Zm00001d033050 | Jasmonate ZIM domain-containing protein (JAZ) |
| Zm00001d048263 | Jasmonate ZIM domain-containing protein (JAZ) |
| Zm00001d048268 | Jasmonate ZIM domain-containing protein (JAZ) |
| Zm00001d017284 | Auxin response factor (ARF) |
| Zm00001d026308 | SAUR family protein (SAUR) |
| Zm00001d014723 | Two-component response regulator ARR-B family (ARR-B) |
| Zm00001d003451 | Ethylene-insensitive protein 3 (EIN3) |
| Zm00001d033267 | Phytochrome-interacting factor 4 (PIF4) |
| Zm00001d039764 | Phytochrome-interacting factor 4 (PIF4) |
| Zm00001d010284 | Gibberellin receptor GID1 (GID1) |
| Zm00001d013099 | Gibberellin receptor GID1 (GID1) |
| Zm00001d038178 | Gibberellin receptor GID1 (GID1) |
| Zm00001d039795 | Gibberellin receptor GID1 (GID1) |
| Zm00001d009626 | Protein phosphatase 2C (PP2C) |
| Zm00001d012401 | Protein phosphatase 2C (PP2C) |
| Zm00001d018178 | ABA responsive element binding factor (ABF) |
| Zm00001d048345 | BR-signaling kinase (BSK) |
| Zm00001d041327 | Protein brassinosteroid insensitive 1 (BRI1) |
| Zm00001d042319 | Protein brassinosteroid insensitive 1 (BRI1) |
| Zm00001d000288 | Auxin-responsive protein IAA (IAA) |
| Zm00001d008749 | Auxin-responsive protein IAA (IAA) |
| Zm00001d018414 | Auxin-responsive protein IAA (IAA) |
| Zm00001d033319 | Auxin-responsive protein IAA (IAA) |
| Zm00001d039624 | Auxin-responsive protein IAA (IAA) |
| Zm00001d040541 | Auxin-responsive protein IAA (IAA) |
| Zm00001d041416 | Auxin-responsive protein IAA (IAA) |
| Zm00001d043515 | Auxin-responsive protein IAA (IAA) |
| Zm00001d045203 | Auxin-responsive protein IAA (IAA) |
| Zm00001d051911 | Auxin-responsive protein IAA (IAA) |
| Zm00001d014690 | Auxin response factor (ARF) |
| Zm00001d010697 | Auxin responsive GH3 gene family (GH3) |
| Zm00001d017397 | SAUR family protein (SAUR) |
| Zm00001d018200 | SAUR family protein (SAUR) |
| Zm00001d039260 | Two-component response regulator ARR-B family (ARR-B) |
| Zm00001d047220 | Serine/threonine-protein kinase SRK2 (SNRK2) |
| Zm00001d028974 | Ethylene-insensitive protein 3 (EIN3) |
| Zm00001d047563 | Ethylene-insensitive protein 3 (EIN3) |
| Zm00001d000408 | EIN3-binding F-box protein (EBF1_2) |
| Zm00001d036880 | EIN3-binding F-box protein (EBF1_2) |
| Zm00001d017778 | Phytochrome-interacting factor 4 (PIF4) |
| Zm00001d051569 | Phytochrome-interacting factor 4 (PIF4) |
| Zm00001d015815 | DELLA protein (DELLA) |
| Zm00001d040536 | Phytochrome-interacting factor 3 (PIF3) |
| Zm00001d010465 | BRI1 kinase inhibitor 1 (BKI1) |
| Zm00001d042833 | Coronatine-insensitive protein 1 (COI-1) |
| Zm00001d002029 | Jasmonate ZIM domain-containing protein (JAZ) |
| Zm00001d002143 | Transcription factor TGA (TGA) |
| Zm00001d024160 | Transcription factor TGA (TGA) |
| Zm00001d038296 | Transcription factor TGA (TGA) |
| Zm00001d016105 | Abscisic acid receptor PYR/PYL family (PYL) |
| Zm00001d007908 | Gibberellin receptor GID1 (GID1) |
| Zm00001d022320 | Gibberellin receptor GID1 (GID1) |
| Zm00001d001865 | Two-component response regulator ARR-A family (ARR-A) |
| Zm00001d025472 | Two-component response regulator ARR-A family (ARR-A) |
| Zm00001d043922 | Auxin response factor (ARF) |
| Zm00001d031665 | Phytochrome-interacting factor 4 (PIF4) |
| Zm00001d045507 | DELLA protein (DELLA) |
| Zm00001d006677 | Brassinosteroid resistant 1/2 (BZR1_2) |
| Zm00001d052323 | Protein brassinosteroid insensitive 1 (BRI1) |
| Zm00001d050861 | Ethylene-insensitive protein 3 (EIN3) |
| Zm00001d009087 | Transcription factor TGA (TGA) |
| Zm00001d004089 | Pathogenesis-related protein 1 (PR1) |
| **Phenylpropanoid biosynthesis** | |
| Zm00001d021324 | Shikimate O-hydroxycinnamoyltransferase (HCT) |
| Zm00001d027946 | Shikimate O-hydroxycinnamoyltransferase (HCT) |
| Zm00001d050455 | Shikimate O-hydroxycinnamoyltransferase (HCT) |
| Zm00001d036933 | Coniferyl-aldehyde dehydrogenase (REF1) |
| Zm00001d017276 | Phenylalanine ammonia-lyase (PAL) |
| Zm00001d017279 | Phenylalanine ammonia-lyase (PAL) |
| Zm00001d051166 | Phenylalanine ammonia-lyase (PAL) |
| Zm00001d013862 | Ferulate-5-hydroxylase,CYP84A (F5H) |
| Zm00001d012980 | Cinnamoyl-CoA reductase (CCR) |
| Zm00001d032152 | Cinnamoyl-CoA reductase (CCR) |
| Zm00001d008435 | Beta-glucosidase (bglB) |
| Zm00001d033649 | Beta-glucosidase (bglB) |
| Zm00001d053982 | Beta-glucosidase (bglX) |
| Zm00001d003317 | Beta-glucosidase |
| Zm00001d013268 | Beta-glucosidase |
| Zm00001d023994 | Beta-glucosidase |
| Zm00001d024000 | Beta-glucosidase |
| Zm00001d016471 | Trans-cinnamate 4-monooxygenase (CYP73A) |
| Zm00001d002004 | Peroxidase (POD) |
| Zm00001d008266 | Peroxidase (POD) |
| Zm00001d036835 | Peroxidase (POD) |
| Zm00001d038599 | Peroxidase (POD) |
| Zm00001d040364 | Peroxidase (POD) |
| Zm00001d011443 | 4-coumarate--CoA ligase (4CL) |
| **Other DEGs related to cell cycle and wuschel homeobox protein** | |
| Zm00001d036371 | Cell division protease FtsH (ftsH, hflB) |
| Zm00001d023511 | Cell division protease FtsH (ftsH, hflB) |
| Zm00001d042285 | Cyclin-dependent kinase 12/13 (CDK12_13) |
| Zm00001d022041 | Cyclin-dependent kinase 12/13 (CDK12_13) |
| Zm00001d041957 | Cell division cycle 20-like protein 1, cofactor of APC complex (CDH1) |
| Zm00001d035270 | Cell division cycle 20-like protein 1, cofactor of APC complex (CDH1) |
| Zm00001d002662 | Cyclin B (CCNB) |
| Zm00001d026129 | Cyclin B (CCNB) |
| Zm00001d043164 | Cyclin B (CCNB) |
| Zm00001d033365 | Cyclin A (CCNA) |
| Zm00001d043444 | Cyclin A (CCNA) |
| Zm00001d028714 | Cyclin A (CCNA) |
| Zm00001d013933 | Cyclin A (CCNA) |
| Zm00001d023283 | Cell division control protein 45 (CDC45) |
| Zm00001d041826 | Cell division cycle 2-like (CDC2L) |
| Zm00001d017128 | Cell division cycle 2-like (CDC2L) |
| Zm00001d048143 | Cyclin D5, plant (CYCD5) |
| Zm00001d035535 | Putative wuschel homeobox protein (WOX) |
| Zm00001d042821 | WUSCHEL-related homeobox 9-like (WOX9) |
