# Supplementary material for: Transcriptome sequencing analysis of maize embryonic callus during early redifferentiation
Source: BMC Genomics. 2019 Feb 27;20:159. doi: 10.1186/s12864-019-5506-7 (PMC6391841; doi:10.1186/s12864-019-5506-7)
Supplement: Supplementary file 20 — Table S1. Plant culture medium formula. (DOCX 14 kb) [file 12864_2019_5506_MOESM20_ESM.docx]

Table S1 Plant culture medium formula

| culture medium kinds | medium component |
| --- | --- |
| Basic media | Ⅰ. N6+ Sugar 30g/L+ Powdered agar 7g/L，pH5.8  Ⅱ. MS+ Sugar 30g/L+ Powdered agar 7g/L，pH5.8 |
| Inducting medium | Basic media I +2,4-D 2mg/L+ Inositol 120mg/L+L-proline 1.38g/L+ Acid hydrolyzed casein 500mg/L |
| Subculture medium | Basic media I +2,4-D 2mg/L+ Inositol 120mg/L+L-proline 0.69g/L+ Acid hydrolyzed casein 100mg/L+ Mannitol 20g/L |
| Differentiation medium | Basic mediaⅡ+KT 1 mg/L+ Acid hydrolyzed casein 100mg/L+ L-proline 0.69g/L |
