# Supplementary material for: Transcriptome sequencing analysis of maize embryonic callus during early redifferentiation
Source: BMC Genomics. 2019 Feb 27;20:159. doi: 10.1186/s12864-019-5506-7 (PMC6391841; doi:10.1186/s12864-019-5506-7)
Supplement: Supplementary file 21 — Table S2. Primers of real-time qRT-PCR assay used in this study. (DOCX 15 kb) [file 12864_2019_5506_MOESM21_ESM.docx]

Table S2 Primers of real-time qRT-PCR assay used in this study

| **Gene** | **Left primer (5’ to 3’)** | **Right primer (5’ to 3’)** |
| --- | --- | --- |
| Zm00001d049641 (*GADPH*) | CCATCACTGCCACACAGAAAAC | AGGAACACGGAAGGACATACCAG |
| Zm00001d041327 | CGGCGAACAGGCTATCTGG | TCGACGTAGTTGAACCCTGG |
| Zm00001d033049 | CGCAGCTGACCATCTCGTA | CTTGCGCCTCTCCATGAAC |
| Zm00001d018178 | GTCATCCTTGTCGCCATCAC | TATGCCTGTTTCCTTTGGCG |
| Zm00001d047789 | GCCGCACTGTTTGACTTCAA | TTCATCTTGTTAAGCGCCGC |
| Zm00001d019518 | CGCTAGCTACCGTGTGATTG | GCAGAGAGCGATTTATGTGCA |
| Zm00001d018157 | CGAAGCCTTGAAGTGGTACG | AGCGAACCCCATCAAGAAGA |
| Zm00001d008230 | TGAATGGTGAAAACCGCTGG | CAAAACTGGAAGGCTCGCTT |
| Zm00001d022041 | ATATCCCAGCACCCTTCGAG | CGTAGCTTTGCGTCCATCTC |
| Zm00001d049387 | TCAAGACCGACAAGCCCTAC | GGAGACCAGTCATCCTCGTT |
| Zm00001d014723 | CGCCATTGGAGGATCACAAG | TCTTGGGCAATCAGGGTCAT |
